# An improved Erk biosensor detects oscillatory Erk dynamics driven by mitotic erasure during early development

**DOI:** 10.1016/j.devcel.2023.08.021

**Published:** 2023-09-09

**Authors:** Scott G. Wilcockson, Luca Guglielmi, Pablo Araguas Rodriguez, Marc Amoyel, Caroline S. Hill

**Affiliations:** 1 Developmental Signalling Laboratory, The Francis Crick Institute, London NW1 1AT, UK; 2 Department of Cell and Developmental Biology, University College London, London WC1E 6BT, UK

## Abstract

Extracellular signal-regulated kinase (Erk) signaling dynamics elicit distinct cellular responses in a variety of contexts. The early zebrafish embryo is an ideal model to explore the role of Erk signaling dynamics *in vivo*, as a gradient of activated diphosphorylated Erk (P-Erk) is induced by fibroblast growth factor (Fgf) signaling at the blastula margin. Here, we describe an improved Erk-specific biosensor, which we term modified Erk kinase translocation reporter (modErk-KTR). We demonstrate the utility of this biosensor *in vitro* and in developing zebrafish and *Drosophila* embryos. Moreover, we show that Fgf/Erk signaling is dynamic and coupled to tissue growth during both early zebrafish and *Drosophila* development. Erk activity is rapidly extinguished just prior to mitosis, which we refer to as mitotic erasure, inducing periods of inactivity, thus providing a source of heterogeneity in an asynchronously dividing tissue. Our modified reporter and transgenic lines represent an important resource for interrogating the role of Erk signaling dynamics *in vivo*.

## Graphical abstract

**Graphic abstract:**
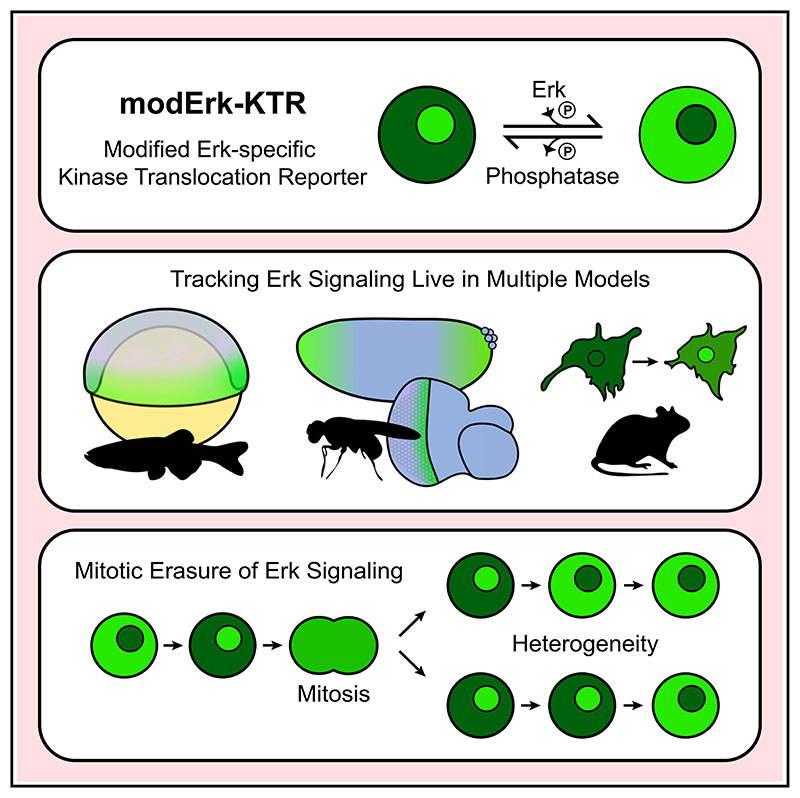

## Introduction

Embryonic development requires coordinated communication, proliferation, and movement of cells on a grand scale. The highly conserved extracellular signal-regulated kinase (Erk) is a key node connecting these processes and plays a critical role in coordinating cell fate specification.^1^ Understanding the regulation and output of Erk signaling is therefore crucial for understanding its role in development as well as in adult homeostasis and disease. Thus, the development of sensitive methods for visualizing signaling dynamics *in vivo* is essential.

Functioning downstream of receptor tyrosine kinase (RTK) receptors, Erk signaling elicits different cellular responses depending on context and upstream ligand-receptor combinations that are associated with distinct signaling dynamics. For example, treatment of rat PC-12 cells with EGF (epidermal growth factor) induces transient signaling, which promotes proliferation, whereas NGF (nerve growth factor) induces sustained signaling that promotes differentiation.^2,3^ Introducing regular pulses of EGF, rather than sustained addition, is sufficient to convert EGF to a pro-differentiation signal.^4^ Similarly, although short-term sustained Erk signaling (~30 min) promotes neural fate in the *Drosophila* blastoderm, long-term sustained (≥60 min) or frequent pulses of Erk activity promote endodermal fate.^5,6^ It appears, therefore, that information is encoded within Erk dynamics through the cumulative dose of Erk activity. The advent of Erk biosensors is now enabling the interrogation of Erk dynamics *in vivo*, and recent work has similarly suggested a role for sustained versus pulsatile signaling in mouse embryonic stem cell (mESC) differentiation,^7,8^ highlighting the importance of elucidating the role of signaling dynamics in regulating cellular identity and behavior.

The zebrafish embryo is an ideal system to study Erk signaling dynamics, and previous studies have successfully employed Erk biosensors to study wounding and vasculogenesis.^9–11^ During early development, the patterns and roles of Erk signaling down-stream of Fgf (fibroblast growth factor) are well characterized, as successive rounds of signaling pattern first the dorsoventral (DV) axis and then the anteroposterior (AP) axis.^12,13^ Between 3.3 and 3.6 h post fertilization (hpf) (mid-blastula stage), a discrete domain of *fgf8a*/*fgf3*/*fgf24* expression and a corresponding diphosphorylated Erk (P-Erk) gradient are induced by Nodal signaling in the presumptive dorsal organizer.^14,15^ Between 4.3 and 5.3 hpf, Nodal signaling induces expression of *fgf8a*/*fgf3*/ *fgf24* in the marginal-most cells to drive long-range Erk signaling around the embryonic margin.^14,16,17^ Together, Nodal and Fgf signaling induce and pattern the mesodermal and endodermal lineages.^18^ Importantly, snap-shot views of development show a highly heterogeneous pattern of Erk activity, as read out by levels of P-Erk.^19^ This pattern suggests heterogeneity in either the single-cell response to Fgf signaling or in Erk signaling dynamics over time.

Here, we report the generation of a highly specific and sensitive reporter of Erk signaling through the modification of the Erk kinase translocation reporter (KTR)^20^ that abolishes the reporter’s responses to cyclin-dependent kinase 1 (Cdk1). We hereafter refer to the biosensor as modErk-KTR. These KTR reporters use site-specific phosphorylation by the target kinase to regulate nucleocytoplasmic shuttling of a fluorescent protein. Thus, KTR readout is sensitive to the activity of both the target kinase and reporter phosphatases. We demonstrate the highly specific sensitivity of the modErk-KTR to Erk signaling in zebrafish embryonic tissues as well as in *Drosophila* embryonic and larval tissues. Furthermore, we monitor the growth and collapse, following inhibition of signaling, of the P-Erk gradient in the zebrafish blastula. We identify oscillations in Erk signaling associated with mitosis, a process we name mitotic erasure, in both zebrafish and *Drosophila* embryos. This introduces periods of Erk inactivity and couples signaling dynamics to tissue growth, thus providing a source of signaling heterogeneity in an asynchronously dividing tissue.

## Design

We observe off-target Erk-KTR activity during early development that we demonstrate is due to Cdk1 activity. Indeed, studies with other Erk biosensors also reported that Cdk1 activity can influence reporter readouts.^21,22^ A recent study addressed this issue with an ERK-specific fluorescence resonance energy transfer (FRET) sensor (EKAREV) by changing the ERK phosphorylation motifs to remove key lysines that mediate Cdk1 recognition.^22^ The phosphorylation sites of the Erk-KTR are similarly surrounded by lysines, but these residues are essential for the function of the nuclear localization sequence (NLS).^20^ It is therefore not possible to modify the Erk-KTR phosphorylation sites to reduce Cdk1 interaction. Instead, we have modified a putative cyclin-docking site found within the ELK1-derived Erk-docking domain to reduce cyclin-Cdk1 binding and hence substantially improve Erk specificity of the biosensor.

## Results

### Erk-KTR displays Mek/Erk-independent off-target activity in early zebrafish embryos

**Figure 1:**
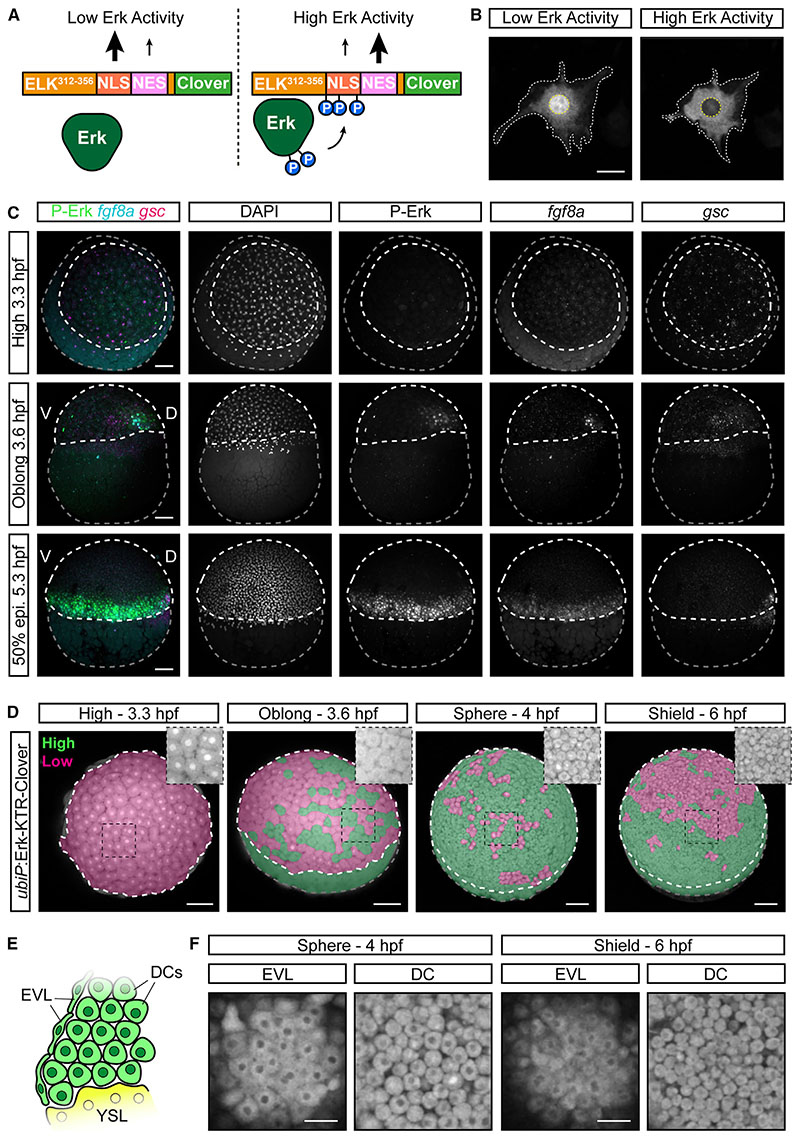
Off-target Erk-KTR activity in the early zebrafish embryo (A) Schematic of the Erk-KTR construct showing the N-terminal Erk-docking domain derived from ELK1, a nuclear localization sequence (NLS) containing Erk-consensus phosphorylation sites (P), a nuclear export sequence (NES) and a C-terminal fluorescent protein, Clover. (B) Live images of an NIH-3T3 cell transfected with *ubiP*:Erk-KTR-Clover construct. The cytoplasmic-to-nuclear ratio of the Erk-KTR fluorescence provides a live readout of relative Erk activity levels. White-dashed line, cytoplasm; yellow-dashed line, nucleus. (C) Combined immunofluorescence and RNAscope showing diphosphorylated Erk (P-Erk) and *fgf8a* expression relative to the dorsal organizer marked by *goosecoid (gsc*) expression. Embryos are oriented in an animal view (3.3 hpf) or lateral view (3.6-5.3 hpf). White-dashed line, embryo proper; gray-dashed line, yolk; 50% epi, 50% epiboly. (D) Stills of live *ubiP*:Erk-KTR-Clover embryos. Embryos are false-colored to indicate Erk-KTR activity as readout by the KTR reporter in a binary manner (green, high activity; magenta, low activity). Embryos are shown from an animal-lateral view. Insets show a magnified view of the boxed region without false coloring. (E) Schematic cross-section of the embryonic margin showing the relative position of the deep cells (DCs), the enveloping layer (EVL) and the yolk syncytial layer (YSL). (F) Single *z*-slices showing Erk-KTR activity in the EVL and DCs from the indicated embryos in (D). Scale bars, 25 μm (B), 50 μm (F), or 100 μm (C and D).

To monitor Erk activity *in vivo*, we used a previously developed transgenic zebrafish line (*ubiP*:Erk-KTR-Clover) where Erk-KTR is ubiquitously expressed.^9^ The Erk-KTR consists of an N-terminal Erk-docking domain (derived from human ELK1), an NLS containing Erk-consensus phosphorylation sites (S/TP motif), a nuclear export sequence (NES), and a green fluorescent protein (Clover) (Figure 1A).^20^ In the absence of activated P-Erk the NLS dominates, and the reporter concentrates in the nucleus (Figures 1A and 1B). Upon Erk activation, phosphorylation of the NLS inhibits its function, and the KTR accumulates in the cytoplasm. By measuring the cytoplasmic-to-nuclear (C/N) fluorescence ratio of the reporter, real-time levels of Erk activity can be measured.^20^

**Figure 2:**
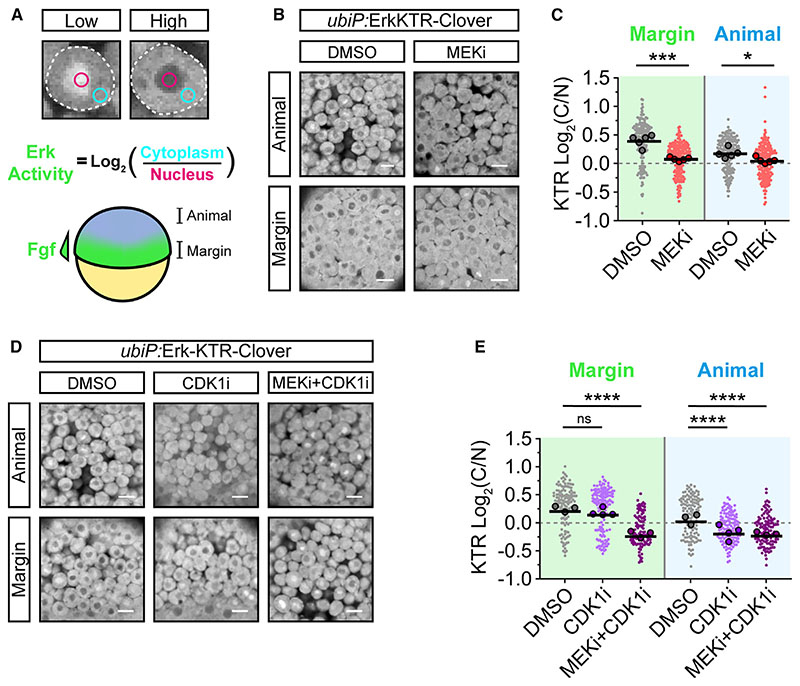
Erk-KTR reports on Erk and Cdk1 activity in early zebrafish embryos (A) Illustration of the method used to report Erk-KTR activity in early zebrafish embryos (schematized below) by measuring mean fluorescence intensity in a region of the nucleus (magenta) and cytoplasm (cyan). The margin of the embryo exhibits high Fgf signaling, while the animal pole does not. (B) Live imaging of *ubiP*:Erk-KTR-Clover transgenic embryos at either the margin or animally, as indicated in (A), following treatment with DMSO (control) or 10 μM PD-0325901 (MEKi) for an hour from 4.0 hpf. (C) Quantification of Erk-KTR activity in (B) at the margin (***p = 0.0002) and animally(*p = 0.0207). n = 178–209 cells per condition from 5 embryos. Shown arethe single-cell readouts of Erk-KTR activity overlayed with the per embryo averages and the overall mean. (D) As in (B) but following treatment with DMSO (control), 20 μM RO-3306 (CDK1i), or both 10 μM PD-0325901 and 20 μM RO-3306 (MEKi + CDK1i) for 1 h from 4.0 hpf. (E) Quantification of Erk-KTR activity in (D) as in (C) for CDK1i (margin p = 0.3131; animal p < 0.0001) or both MEKi and CDK1i (margin p < 0.0001; animal p < 0.0001). n = 107–146 cells per condition from 3 (DMSO and MEKi + CDK1i) or 4 embryos (CDK1i) per condition. Statistical tests were Student t test (C) or one-way ANOVA with Sidak’s multiple comparisons test (E). Scale bars, 20 μm; **** p < 0.0001; ns, not significant.

First, we established whether Erk-KTR reports the known patterns of P-Erk in the zebrafish blastula. P-Erk is first seen at 3.6 hpf in a dorsal domain colocalizing with *gsc* and *fgf8a* expressions (Figure 1C). By 5.3 hpf, P-Erk is detected throughout the embryonic margin in both deep cells (DCs) and the enveloping layer (EVL).^16^ To aid visualization of the Erk-KTR readout, we false-colored embryos in a binary manner to show enrichment in the cytoplasm (green; C:N > 1) or nucleus (magenta; C:N ≤ 1), indicating high and low Erk activities, respectively (Figure 1D). At 3.3 hpf, the reporter shows strong nuclear localization throughout the blastoderm, indicating no Erk activity (Figure 1D). However, from 3.6 hpf, we observed sporadic nuclear exclusion throughout the blastoderm, and most cells showed uniform nuclear exclusion by 4.0 hpf, including both DCs and the EVL (Figures 1E and 1F). By 6.0 hpf, nuclear exclusion of the reporter becomes restricted to the margin, but it was still observed beyond the Fgf/Erk signaling domain (Figure 1D; Video S1). To test whether Erk-KTR localization was Erk dependent, we measured C/N ratios at both the margin (high Fgf/Erk) and animal pole (no Fgf/Erk; Figure 2A) following treatment from 4.0 to 5.0 hpf with an inhibitor of Mek (mitogen-activated protein kinase kinase), the upstream activator of Erk (10 μM PD-0325901; MEKi). This caused a small but significant decrease in C/N ratios at both the margin and animal pole compared with controls (Figures 2A-2C). However, Erk-KTR remained enriched in the cytoplasm in treated embryos, indicating significant Mek/Erk-independent activity.

Previous studies have used the Erk-KTR to monitor signaling dynamics at later stages of development (≥24 hpf),^9,11^ and we observed that by 6.0 hpf, the reporter more accurately reflected the expected pattern of Erk signaling (Figure 1D). This suggested that the Mek/Erk-independent localization might be driven by some phenomenon occurring during early development. We noted that the onset of reporter mislocalization correlated well with cell cycle remodeling at the midblastula transition.^23,24^ This is driven by lengthening of the cell cycle, which is influenced by changes in Cdc25-Cdk1 activity in zebrafish.^23^ We therefore investigated whether Erk-KTR localization was influenced by cell cycle inputs by treating cells with a CDK1-specific inhibitor (20 μM RO-3306; CDK1i). Treatment had no effect on Erk-KTR C/N ratios at the margin; however, there was a significant reduction in C/N ratios animally (Figures 2D and 2E). Treatment with both MEKi and CDK1i resulted in maximal nuclear localization at the margin. Together, these data show that the localization of Erk-KTR is controlled by a combination of Erk and Cdk1. We propose that the short cell cycles (every 15–30 min) of early development emphasize this due to an increased frequency of high Cdk1 activity, masking the true pattern of Erk signaling.

### modERK-KTR: a modified Erk-KTR with improved specificity

**Figure 3:**
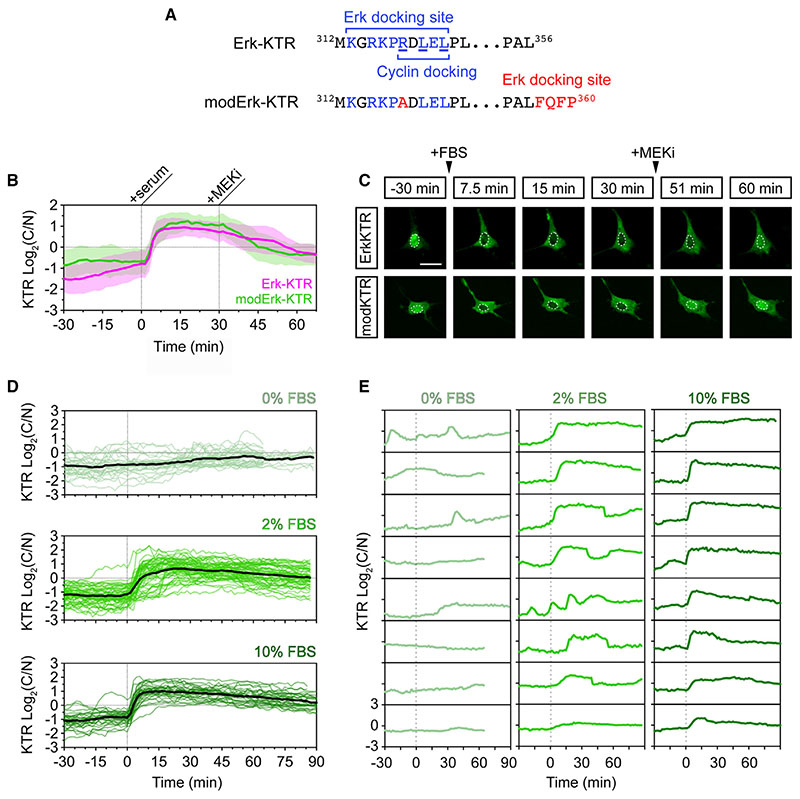
A modified Erk-KTR reports ERK activity in mouse fibroblasts (A) Amino acid sequences of the Erk-docking domain of Erk-KTR and modified Erk-KTR (modErk-KTR) highlighting the modifications (red) made to reduce off-target reporter activity, including the R>A substitution within the docking site and the additional FQFP Erk-docking site between the ELK fragment and the NLS. (B) Quantification of ERK activity in NIH-3T3 cells transfected with the Erk-KTR or modErk-KTR constructs (n = 58 cells each, mean ± SD). Cells were serum-starved overnight and ERK was induced by the addition of 10% FBS. ERK activity was inhibited after 30 min with 10 μM PD-0325901 (MEKi). (C) Representative images of reporter activity in (B). (D) Quantification of ERK activity in NIH-3T3 cells transfected with the modErk-KTR construct after overnight serum starvation, followed by the addition of different concentrations of FBS. Individual cell traces and the mean (black line) are shown for 0% FBS (n = 32 cells), 2% FBS (n = 57 cells), and 10% FBS (n = 30 cells). (E) Individual cell traces from (D). Scale bars, 20 μM.

To specifically monitor Fgf/Erk signaling dynamics *in vivo*, we generated an improved Erk biosensor devoid of Cdk1 responsiveness (modErk-KTR). To achieve this, we introduced an R>A substitution within a putative cyclin-docking site (RxLxΦ, where Φ is a hydrophobic residue) in the Erk-docking domain (Figure 3A).^25^ This is predicted to significantly reduce cyclin-substrate binding^26,27^ but may also weakly reduce Erk binding.^28,29^ We therefore introduced another four amino acid Erk-docking site (FQFP) at the C terminus of the ELK1 fragment (Figure 3A).^29^

We initially characterized modErk-KTR in NIH-3T3 mouse fibroblasts to ensure the modifications had not compromised the biosensor’s Erk sensitivity.^20^ NIH-3T3 cells divide infrequently, particularly with serum starvation, and thus display minimal Cdk1-dependent effects on KTR localization.^30^ Following overnight serum starvation, cells displayed a baseline low C/N ratio indicative of low/no ERK activity (Figures 3B and 3C). Upon addition of 10% fetal bovine serum (FBS), a rapid increase in C/N ratio was observed for both biosensors within ~5 min, which was inhibited within 15–30 min with MEKi (10 μM PD-0325901) (Figure 3C; Video S2). We also observed a serum concentration-dependent response of modErk-KTR (Figures 3D and 3E). Addition of 10% FBS elicited a rapid, sustained increase in C/N ratios over 1.5 h, whereas 2% FBS elicited a slower response with reduced amplitude as well as more transient and oscillatory dynamics (Figure 3E). In uninduced samples, the baseline C/N ratio was sustained over a similar time course with some low-level sporadic activity (Figure 3E). These data show that the reporter modifications have not affected the Mek/Erk-dependent response and demonstrate that modErk-KTR captures the full range of Erk signaling dynamics previously described with Erk-KTR *in vitro*.

**Figure 4:**
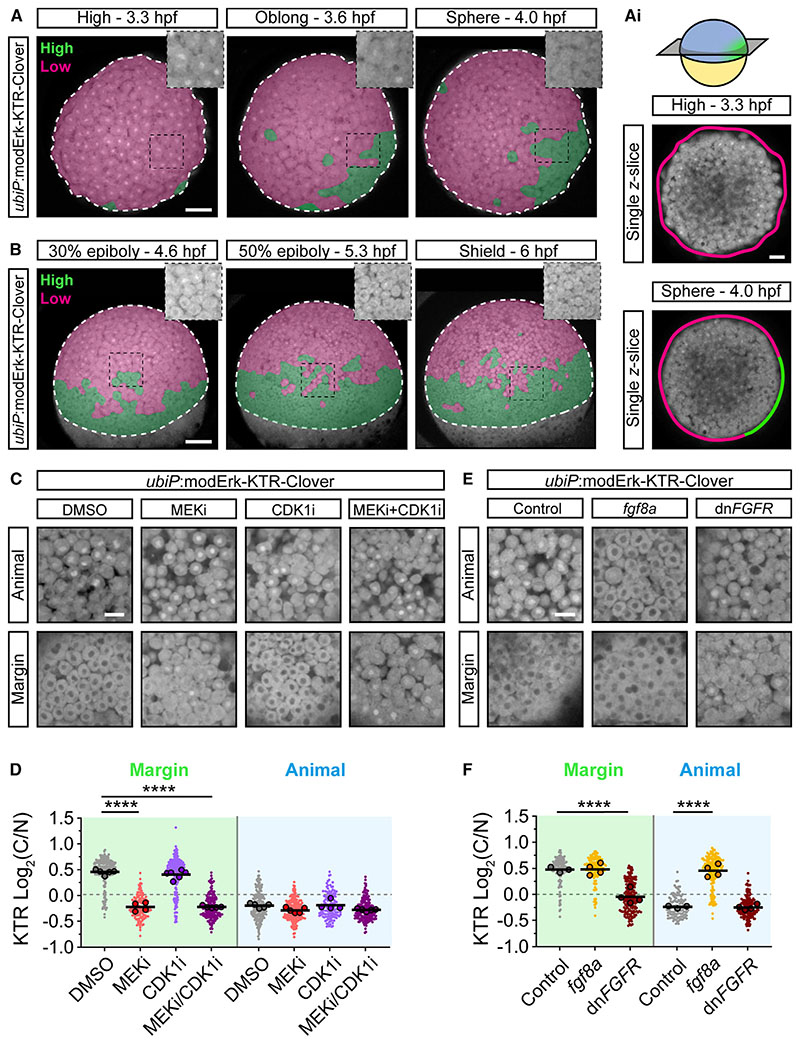
modErk-KTR specifically reports on Fgf/Erk activity in early zebrafish embryos (A and B) Stills of live *ubiP*:Erk-KTR-Clover transgenic embryos. Embryos are false-colored to indicate Erk activity levels; (green, high activity; magenta, low activity). Embryos are shown from an animal (A) or lateral (B) view. Insets show a magnified view of the region within the black box without false coloring. White-dashed line, embryo proper. (Ai) Single *z*-slices through the center of embryos in (A) showing Erk activity around the embryonic margin using the same color scheme as (A). (C) Live imaging of *ubiP*:Erk-KTR-Clover transgenic embryos following treatment with DMSO (control), 10 μM PD-0325901 (MEKi), 20 μM RO-3306 (CDK1i), or both MEKi and CDK1i for an hour from 4.0 hpf. (D) Quantification of Erk activity in (C) at the margin and animally. Shown are the single-cell readouts of Erk activity overlayed with the per embryo averages and the overall mean for DMSO (control, n = 209 cells from 5 embryos [margin] or n = 200 cells from 5 embryos [animal]), 10 μM PD-0325901 (MEKi; n = 137 cells from 4 embryos [margin] or n = 196 from 5 embryos [animal]), 20 μM RO-3306 (CDK1i; n = 227 cells from 6 embryos [margin] or n = 133 cells from 4 embryos [animal]) or both 10 μM PD-0325901 and 20 μM RO-3306 (MEKi + CDK1i; n = 183 cells from 5 embryos [margin] or n = 194 cells from 5 embryos [animal]) treated embryos. (E) Live imaging as in (C) of embryos injected with either 25 pg *fgf8a* or 500 pg *dnFGFR* at one-cell stage. Embryos were imaged at 50% epiboly (5.3 hpf). (F) Quantification of Erk activity in (E) as in (D) for control (n = 142 cells from 3 embryos [margin] and n = 116 cells from 3 embryos [animal]), *fgf8a* (n = 130 cells from 4 embryos [margin], or n = 192 cells from 4 embryos [animal]) or *dnFGFR* (n = 168 cells from 4 embryos [margin] or n = 157 cells from 4 embryos [animal]) injected embryos. Statistical tests were one-way ANOVA with Šidák’s multiple comparisons test. Scale bars, 100 μm (A and B), 50 μm (Ai) or 20 μm (C–E); **** p > 0.0001. See also Figure S1.

Next, we tested the functionality of modErk-KTR *in vivo* by generating *Tg*(*ubiP*:modErk-KTR-Clover) transgenic zebrafish and investigating whether modErk-KTR faithfully recapitulated the DV and AP axis P-Erk patterns using the binary classification described above (Figure 1D). At 3.3 hpf, the reporter showed strong nuclear localization throughout the blastoderm, indicating no Erk activity (Figure 4A), similar to Erk-KTR (Figure 1D). From 3.6 hpf, unlike Erk-KTR, the high C/N ratios of modErk-KTR were restricted to a discrete domain in marginal cells, representing the presumptive dorsal organizer (Figures 4A and 4Ai; Video S3). From 4.6 hpf, this domain of high C/N ratios expanded to encompass the embryonic margin, including both DCs and EVL (Figure S1A), with very few animal cells showing high C/N ratios (Figure 4B; Video S4). In addition, the width of the gradient of high C/N ratios progressively expanded from 4.6 to 5.3 hpf but remained limited to the marginal cells up until 6.0 hpf. In summary, a qualitative view of zebrafish blastulae shows that modErk-KTR recapitulates the expected patterns of Fgf/Erk activity.

To address whether the observed reporter activity was solely Mek/Erk dependent, we monitored KTR localization at the margin and animal pole following treatment with MEKi, CDK1i, or both (Figures 4C and 4D). Treatment from 4.0 hpf for 1 h with MEKi caused a significant decrease in the C/N ratio at the margin compared with the control but had no effect on cells at the animal pole. Conversely, we observed no effect on reporter localization following the addition of CDK1i, whereas MEKi/CDK1i together led to a similar decrease in the C/N ratio as MEKi alone. These data suggest that the KTR modifications have successfully extinguished the Cdk1 sensitivity in zebrafish embryos.

To further confirm the functionality of modErk-KTR as a readout of Fgf signaling, we ubiquitously overexpressed Fgf8a or dominant negative Fgf receptor (dnFgfR) and monitored KTR localization (Figures 4E and 4F). Overexpression of Fgf8a resulted in no further increase in C/N ratio in the most marginal cells but induced a significant increase animally, with embryos showing uniformly high C/N ratios (Figure 4F). Conversely, over-expression of dnFgfR significantly reduced C/N ratios in the most marginal cells, consistent with the reduction in P-Erk levels shown previously,^16^ while having no effect on cells at the animal pole. modErk-KTR is therefore a faithful readout of Fgf/Erk signaling in the zebrafish blastula.

We next tested the utility of modErk-KTR in other *in vivo* contexts. Previous studies have used the Erk-KTR to monitor Erk responses in muscle cell wounding at 48 hpf.^9,11^ Importantly, multinucleated muscle cells are post-mitotic and therefore free of Cdk1-dependent influence on reporter localization.^31^ Wounding assays were performed in both Erk-KTR and modErk-KTR transgenic embryos, and their Erk-dependent responses were compared. At homeostasis, muscle cells at 48 hpf did not display any Erk activity (Figure S1B), but rapid cytoplasmic localization of both reporters was observed in cells surrounding the wound within 15 min. Therefore, in this context, the two reporters appear to function similarly.

We also compared the two reporters in other developing tissues exhibiting well-characterized Fgf signaling. modErk-KTR displayed clear nuclear exclusion in the developing eye and tail-bud presomitic mesoderm (Figure S1C). Fgf ligands (e.g., *fgf3*) are discretely expressed at the midbrain-hindbrain boundary (MHB) of 24 hpf embryos (Figure S2A), whereas Fgf target genes (e.g., *pea3*, *erm1*, and *sprouty4*) are expressed in broader domains, suggesting that secreted Fgf ligands act at some distance from their source.^32,33^ Using Erk-KTR, we observed generally high C/N ratios throughout the midbrain and only observed a minimal reduction at 200 μm away from the MHB (Figures S2B and S2D). By comparison, modErk-KTR read out a steeper gradient with a stepwise feature of C/N ratios; the highest C/N ratios were observed at the MHB, with a plateau at 75–150 μm before decreasing again at 150–200 μm (Figures S2C and S2D).

In conclusion, modErk-KTR displays improved Erk specificity in zebrafish embryos and can be used to monitor Erk signaling in a wide variety of developmental contexts *in vivo*.

### An improved reporter system for *Drosophila* embryonic and larval tissues

Erk-KTR was recently adapted for use in *Drosophila* and used to monitor ERK activity in several larval and adult tissues.^34^ It was further developed to include a histone marker (Histone 2Av [H2Av]-mCherry) produced from the same coding sequence but separated by a self-cleaving T2A peptide.^35^ The presumed equimolar concentrations of H2Av/KTR enable the readout of ERK activity by nuclear fluorescence alone in contexts where cells are densely packed and the measurment of cytoplasmic fluorescence is difficult.^34,35^ We thus investigated whether modErk-KTR would offer improved specificity in *Drosophila*, particularly during early development, where reporter localization could be influenced by rapid cell cycles. We generated transgenic lines expressing mod-ERK-KTR and H2Av-mCherry (modERK-KTR-Clover-T2A-H2Av-mCherry) under the control of a *UAS* or *nanos* promoter (*nosP*) for tissue-specific and maternal expressions, respectively. We also generated a transgenic line with ERK-KTR-Clover-T2A-H2Av-mCherry under the control of *nosP* for comparison.

**Figure 5:**
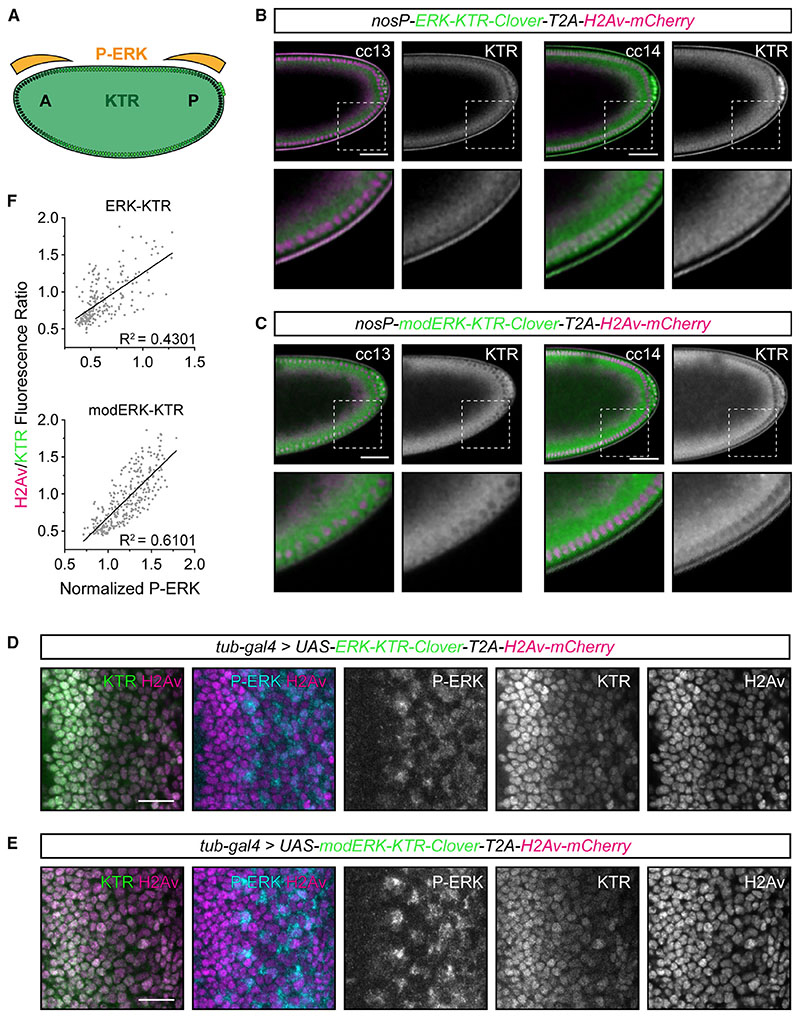
Improved Erk activity reporting with modERK-KTR in ***Drosophila*** embryos and larval tissue (A) Schematic of anteroposterior Torso/ERK signaling during early *Drosophila* development where signaling is restricted to both poles of the blastoderm (shown by orange gradient), excluding the pole cells (cluster of green cells on the right-hand side). Cells shown with black nuclei have high ERK signaling, whereas those with green nuclei have no ERK signaling. (B and C) Representative images of the posterior half of transgenic *Drosophila* embryos maternally expressing the original ERK-KTR (B) or modERK-KTR (C) constructs with a polycistronic H2Av-mCherry tag during cell cycles (cc) 13 and 14. Shown is a single *z*-slice through the center of the embryo (top) and a magnified view of the regions indicated by white boxes (bottom). (D and E) Representative images of eye imaginal discs ubiquitously expressing ERK-KTR (D) or modERK-KTR (E) constructs with a polycistronic H2Av-mCherry tag under the control of *tub-Gal4*. The levels of ERK activity, as read out by ERK-KTR constructs, are here compared with the levels of P-ERK. (F) Quantification of (D) and (E) comparing P-ERK levels with the read out of ERK-KTR (n = 222 cells from 3 discs) or modERK-KTR (n = 293 cells from 3 discs) constructs and fitted with a simple linear regression. See also Figures S3 and S4.

RTK signaling through Torso induces gradients of P-ERK at the anterior and posterior poles of the *Drosophila* blastoderm embryo, excluding the pole cells (Figure 5A).^36^ To compare the KTR readouts of these signaling gradients, we imaged embryos from cell cycle 13 to 14 (Figures 5B, 5C, S3A, and S3B). For a reporter that accurately reads out ERK activity, nuclear exclusion at the poles and nuclear accumulation medially should be observed (Figure 5A). However, ERK-KTR shows low-level nuclear accumulation throughout the length of the embryo with nuclear exclusion at the poles during cell cycle 13 (Figures 5B and S3A), suggesting that ERK-KTR does not accurately read out ERK activity, possibly due to continuously high levels of CDK1 activity (Figure 1D).^37^ By cell cycle 14, coincident with significant lengthening of the cell cycle, ERK-KTR localization was consistent with the pattern of ERK activity: nuclear accumulation of ERK-KTR was evident in mediolateral regions, whereas it was excluded from the nucleus in cells at both poles (Figures 5B, S3A).^38^ By contrast, during both cell cycle 13 and 14, modERK-KTR displayed the predicted pattern of nuclear exclusion at both poles and nuclear accumulation medio-laterally (Figures 5C and S3B; Video S5). This suggests that mod-ERK-KTR represents a substantial improvement in monitoring ERK signaling during *Drosophila* embryonic development.

We also tested whether modERK-KTR offered any improvement in third instar eye imaginal discs, where EGF-ERK activity regulates the differentiation of photoreceptors as cells pass through the morphogenetic furrow.^39^ To visualize the KTRs in eye imaginal discs, we drove ubiquitous transgene expression with *tubulin-Gal4*, performed immunostaining for P-ERK (Figures 5D and 5E), and compared P-ERK levels and H2Av/KTR ratios for individual cells. We found a positive correlation for ERK-KTR (R^2^ = 0.4301), as increasing levels of P-ERK correlated with a higher H2Av/KTR ratio (Figure 5F). However, the correlation between P-ERK levels and H2Av/KTR ratios for mod-ERK-KTR showed an improved linear relationship (R^2^ = 0.6101). Cells in the eye disc are arrested in G1 phase in the morphogenetic furrow before some cells re-enter the cell cycle. Therefore, by examining cells just posterior to the furrow and labeling S-phase cells via 5-ethynyl-2′ -deoxyuridine (EdU) incorporation, we directly compared KTR localization in G1 versus S-phase cells. We focused on P-ERK-negative cells, in which the KTR should be predominantly localized to the nucleus. Using ERK-KTR, we observed cells with similarly low levels of P-ERK that varied in KTR localization: EdU-positive cells (Figure S4A; white-dashed lines) had lower nuclear KTR fluorescence than P-ERK-negative/EdU-negative neighboring cells (yellow-dashed lines). Thus, the cell cycle stage of cells influenced ERK-KTR localization. By contrast, modERK-KTR displayed similar nuclear enrichment in cells that were P-ERK-negative, irrespective of cell cycle phase (Figure S4A). To further examine the cell cycle dependence of ERK-KTR, we compared the readout of the reporters in the adult ovarian germline, as ERK signaling is restricted to surrounding somatic cells.^40^ Early germ cells therefore provide a proliferative but P-ERK-negative background in which the KTRs should localize exclusively to the nucleus. Both reporters showed similar degrees of nuclear accumulation in egg chamber germ cells (Figure S4B). However, the early germ cells within the germarium showed significantly higher H2Av/KTR ratios for ERK-KTR compared with modERK-KTR (Figures S4C and S4D), demonstrating ERK-independent activity of ERK-KTR.

Together, these data show that modERK-KTR provides an improved readout of ERK activity in *Drosophila*, highlighting the fact that cell cycle dependence of ERK-KTR localization is not restricted to zebrafish and should be considered in all proliferative cells/tissues.

### Growth of the Fgf/Erk signaling gradient in zebrafish presumptive mesendoderm

**Figure 6:**
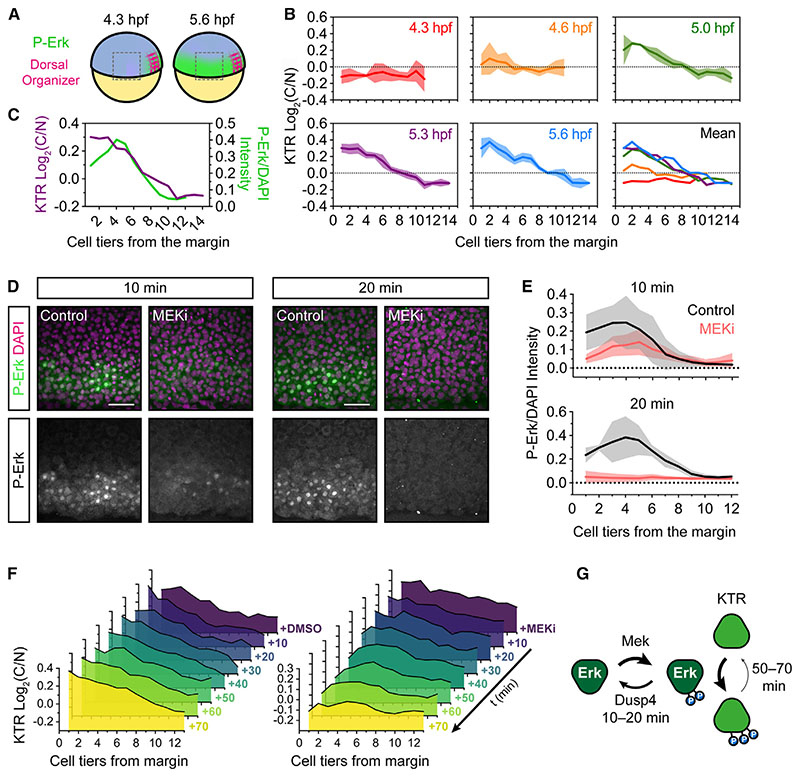
modERK-KTR reads out Fgf/Erk signaling gradient formation in real time (A) Schematic of the zebrafish embryo illustrating the lateral region imaged in (B) relative to the dorsal organizer (see Figure 1C). (B) Quantification of Erk activity (log_2_(C/N)) in the lateral region of *ubiP*:modErk-KTR-Clover embryos at 20 min intervals from dome (4.3 hpf) to germ ring stage (5.6 hpf). Cells were binned based on their distance in cell tiers from the embryonic margin (0). n = 3 embryos showing the per embryo mean ± SD. Also shown is an overlay of the mean levels at each time point. (C) Overlay of the mean Erk activity (modErk-KTR) and P-Erk levels in similarly staged embryos (5.3 hpf). (D) Representative immunofluorescence images of embryos treated with DMSO (control) or MEKi (10 μM PD-0325901) for 10–20 min from 5.0 hpf before fixation. (E) Quantification of P-Erk levels from (D) in cell tiers relative to the embryo margin (0) showing mean ± SD. DMSO 10 min, 5 embryos; MEKi 10 min, n = 6 embryos; DMSO 20 min, n = 4 embryos; MEKi 20 min, n = 4 embryos. (F) Quantification of modErk-KTR read out following treatment with DMSO (control) or 10 μM PD-0325901 (MEKi). Shown is the mean of n = 3 embryos per time point. (G) Schematic comparing the dephosphorylation rates of Erk and its targets. Scale bars, 50 μm. See also Figure S5.

We next asked whether we could track the growth of the Fgf/Erk signaling gradient at the blastula margin in the presumptive mes-endoderm.^18^ We imaged the lateral region of *Tg*(*ubiP*:modErk-KTR-Clover) embryos from 4.3 hpf, when Erk activity is restricted to the dorsal organizer, and then every 5 min for 1.5 h (Figure 6A). C/N ratios were measured relative to distance from the margin and presented at 20-min intervals (Figure 6B). Cells were binned into cell tiers (yolk syncytial layer [YSL] = 0), and we found that during this period of development, cells undergo a change in size due to proliferation (from 24 to 18 μm width; Figures S5A and S5B). Thus, the size of a single-cell tier reduces over time. At 4.3 hpf, there was little to no cytoplasmic enrichment observed, as expected, but by 4.6 hpf, the first 4 cell tiers (~100 μm) from the margin began to show higher C/N ratios (Figure 6B). We noted that the first cell tier (~25 μm) exhibited lower C/N ratios in comparison with cells further from the margin. By 5.0 hpf, the gradient had expanded to eight cell tiers (~175 μm; Figure S5C), and by 5.3 hpf, the full 10-cell tier (~200 μm; Figure S5C) gradient had formed (Figure 6B). We observed a high degree of variability in Erk activity across the gradient and at each time point (Figure S5D). This shows that the response to Fgf signaling is heterogeneous, which is supported by our recent work showing heterogeneity in P-Erk levels.^19^

We found that the lower levels of P-Erk in the first three cell tiers, driven by the activity of the dual-specificity phosphatase Dusp4, were not read out by modErk-KTR by 5.3 hpf (Figure 6C).^18^ This is not likely due to the sensitivity of modErk-KTR, as equivalently low levels of P-Erk are read out in cell tier 6. A more likely explanation is that cell tiers 1–4 have experienced Erk activity for 20 min longer than cells further from the margin, with modErk-KTR reading out the cumulative dose of Erk activity over time. To test this, we monitored the rate of Erk deactivation (comparing P-Erk levels and modErk-KTR localization) following the addition of MEKi from 5.0 hpf. This rate will be determined by the relative activity of both P-Erk and reporter phosphatases. We observed that the majority of P-Erk was dephosphorylated within 10 min, and after 20 min, it was completely extinguished relative to the DMSO control (Figures 6D and 6E).^18^ Cell tiers 1–2 were most sensitive to MEKi and lost P-Erk within 10 min (Figure 6E). Next, we monitored the rate of modErk-KTR deactivation. Following the addition of DMSO at 5.0 hpf, the gradient of KTR C/N localization built up gradually over time, with the highest Erk C/N ratios at the margin (Figures 6F and S5E). In contrast to P-Erk, after the addition of MEKi, the gradient remained unchanged after 20–30 min, and it was only after 40 min that cell tiers 1–2 showed a decreased C/N ratio and after 60–70 min that cell tiers 3–10 showed a decreased C/N ratio (Figures 6F and S5E). This demonstrated that the rate of modErk-KTR dephosphorylation was substantially slower than that of P-Erk in zebrafish blastulae (Figure 6G). We also noted that this is slower than that observed in NIH-3T3 cells (~30 min; Figure 3B) and propose that slow KTR dephosphorylation is an inherent property of the zebrafish embryo. This suggests that modErk-KTR reads out the cumulative dose of Erk activity in the first 1–3 cell tiers rather than absolute levels (Figure 6C). Nevertheless, the lower levels of P-Erk sensitize these cells to changes in upstream signaling.

A recent study identified the dual-specificity phosphatase, calcineurin, as a modulator of FGF/ERK signaling and ERK-KTR read out.^41^ We therefore treated *Tg*(*ubiP*:modErk-KTR-Clover) embryos with the calcineurin inhibitor, cyclosporin A (CsA), to determine if it regulates Erk activity in the presumptive mesendoderm. Incubation with CsA for 24 h is sufficient to drive cardiacedema by 48 hpf (Figure S5F).^42^ However, there was no effect on the average Erk signaling levels at 50% epiboly following a 1.5-h incubation (Figures S5G and S5H), suggesting that calcineurin is not a key phosphatase regulating Fgf signaling in this context.

These data demonstrate the utility of modErk-KTR as a live readout of Erk activity during embryonic development, allowing the visualization of the evolving Fgf signaling gradient *in vivo*.

### Mitotic erasure induces oscillatory Fgf/Erk signaling dynamics

**Figure 7:**
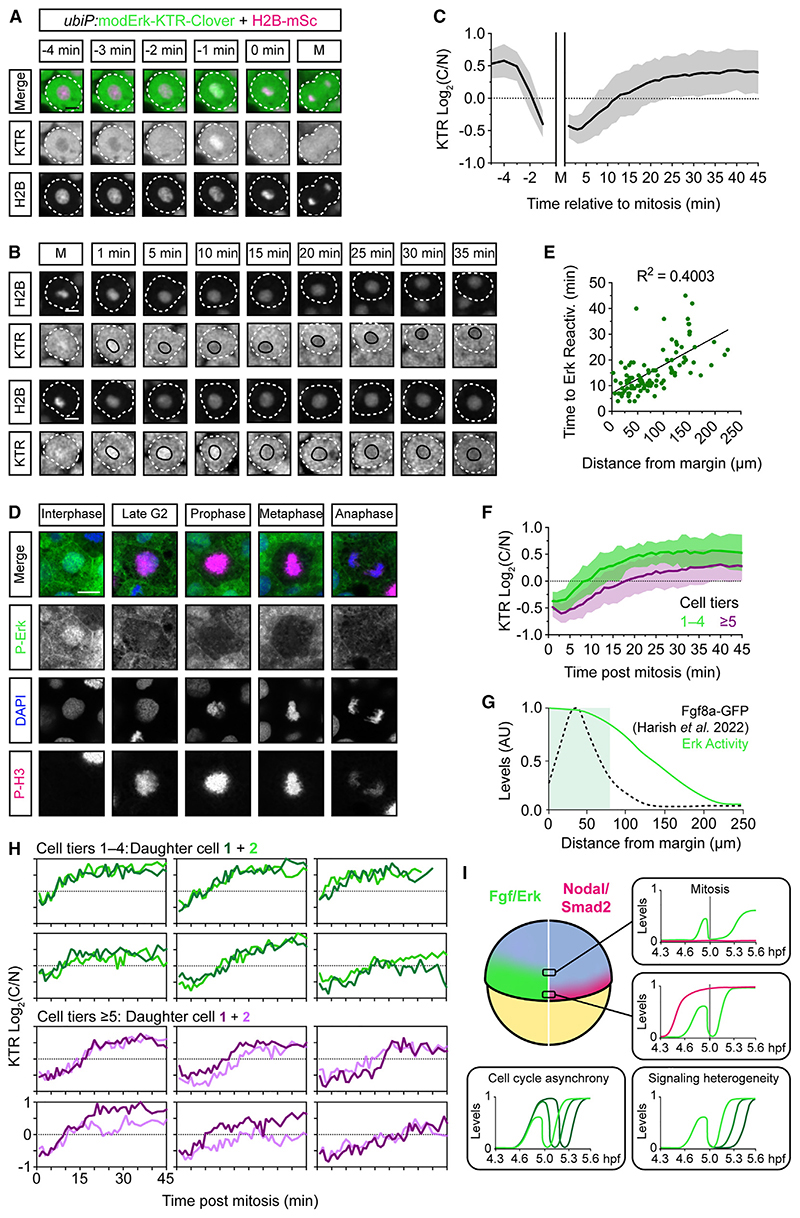
Mitotic erasure induces oscillatory Fgf/Erk signaling dynamics in the presumptive mesendoderm (A) Representative images of a single mesendodermal cell approaching mitosis. *ubiP*:modErk-KTR-Clover embryos were injected with 25 pg *H2B-mScarlet-I* mRNA at the one cell stage and a lateral region of the margin was imaged from ~4.6 hpf at 1 min intervals. White-dashed line labels the single cell. (B) As in (A) following two cells post-mitosis. Black line labels the nucleus. (C) Quantification of Erk activityfrom (A) and (B) following mother cells (n = 56 cells) from −5 min before mitosis and daughter cells (n = 110) +45 min after mitosis showing mean ± SD. Nuclear envelope breakdown means the KTR cannot read out Erk activity during mitosis itself. (D) Representative immunofluorescence images of P-Erk and P-H3 in zebrafish embryos (4.6 hpf). (E) Quantification of the time to Erk reactivation (log_2_(C/N) ≥ 0.25) post-mitosis and the distance of each cell from the embryonic margin (n = 110) and fitted with a simple linear regression. (F) Comparison of Erk reactivation rates from (C) with cells binned based on their initial distance from the margin. (G) Schematic of the Erk activity gradient, as read out by modErk-KTR, and the extracellular levels of Fgf8a-GFP described in similarly staged embryos (~5.3 hpf).^43^ (H) Single-cell traces of sister cells post-mitosis from (E). (I) Model depicting how mitotic erasure of P-Erk and its target proteins induces signaling oscillations. Both the rate of reactivation post-mitosis and the final amplitude of Erk activity are sensitive to a cell’s relative position within the Fgf signaling gradient. Coupled with cell cycle asynchrony and variability in reactivation rates, mitotic erasure introduces heterogeneity to Fgf/Erk signaling in the presumptive mesendoderm. Different green lines correspond to P-Erk levels in different cells. Scale bars, 10 μm. See also Figures S6 and S7.

Heterogeneity in Fgf/Erk signaling is apparent during zebrafish mesendodermal patterning both at the level of P-Erk^19^ and downstream Erk activity (Figure S5D). To address how this arises, we injected one-cell-stage embryos with *H2B-mScar-let-I* mRNA and tracked individual nuclei from ~4.6 hpf. We observed that as cells approached mitosis, there was a rapid (within 2–3 min) decrease in C/N ratio (Figures 7A and 7C; Video S6). Post-mitosis, daughter cells initially displayed low C/N ratios, which increased over time, indicating that they reactivate signaling, but with variable kinetics (Figures 7B and 7C). Intrigu-ingly, we observed this same phenomenon in *Drosophila* at cell cycle 13 (Figure S6A; Video S7). We confirmed that this is not a reporter artifact, as we also observed a loss of P-Erk in phospho-histone H3 (P-H3)-positive cells at the zebrafish margin (Figures 7D and S7B). This is specific to Fgf/Erk signaling, as Nodal-driven P-Smad2 can be maintained throughout mitosis (Figures S7A and S7B). To verify that the KTR nucleocytoplasmic shuttling reflects the phosphorylation-dephosphorylation kinetics of the reporter, we generated non-phosphorylatable and phospho-mimetic variants by mutation of the three phosphorylation sites (STT residues) within the NLS to alanines (AAA) or glutamates (EEE), respectively. One-cell embryos were injected with *modErk-KTR-Clover* variant and *H2B-mScarlet-I* mRNA, and the Fgf/Erk signaling gradient was measured at 5.3 hpf. modErk-KTR^AAA^ displayed consistent nuclear localization, whereas modErk-KTR^EEE^ was constitutively cytoplasmic (Figures S7C and S7D), further illustrating that Erk-dependent phosphorylation of modErk-KTR dictates reporter read out in the presumptive mesendoderm. We tracked individual cells pre-mitosis at similar stages and found that neither mutated variant changed subcellular localization 2–3 min prior to mitosis (Figures S7E-S7H), compared with modErk-KTR. Some nuclear modErk-KTR^EEE^ was observed 1 min prior to mitosis, likely due to initiating nuclear envelope breakdown and the leaking of cytoplasmic reporter into the nucleus. Together, these data reveal that mitotic erasure induces periods of Erk inactivity, which is reflected by rapid KTR relocalization.

We next asked what might dictate the high degree of variability in the rate of post-mitotic reactivation (5–45 min to log_2_(C/ N) > 0.25) and the final amplitude of Erk activity. There was no clear correlation between the Erk levels of the mother cell (−4 min) and their daughter cells (+30 min) (Figure S6B), likely due to the growth of the signaling gradient during this period. There was, however, a positive correlation between sister cells (Figures S6C–S6E). However, this appears to reflect temporal Erk dynamics rather than actual levels (i.e., both sisters start with low C/N ratios and both increase over time). However, post-mitotic reactivation rates positively correlated with a cell’s distance from the margin (R^2^ = 0.4003; Figure 7E), with cells in cell tiers 1–4 reactivating fastest with higher final levels of Erk activity (Figure 7F). The extracellular distribution of endogenous Fgf8a-GFP at the embryonic margin was recently described to be concentrated around cell tiers 1–4 (Harish et al.^43^) (Figures 7G and S6G). Taken together with our data, this suggests that rapid post-mitotic reactivation correlates with extracellular ligand availability.

Despite the general trend toward faster reactivation rates in cell tiers 1–4 (5–20 min) versus 5–10 (10–45 min), we still observed variability between both neighboring cells and sister cells (Figures 7H and S6C–S6E), which increased the further from the margin cells were located (Figures 7E, 7F, and 7H). In addition, we noticed that cells >4 cell tiers away from the margin appear more mobile and traverse entire cell tiers. We therefore asked whether the final location of sister cells at +30 min post-mitosis might explain the variability in Erk activity between sister cells (Figure S6F). Indeed, those sister cells that move away from the margin exhibit lower levels of Erk activity. However, this is only apparent when sisters are separated by more than a single-cell tier (>20 μm). This not only highlights the sensitivity of postmitotic reactivation rate to a cell’s relative position within the Fgf signaling gradient (Figures 7F, 7G, and S6G) but also suggests a degree of cell-autonomous heterogeneity as neighboring sister cells can display different reactivation rates and amplitudes of Erk activity (Figures 7H and S6C–S6E).

In conclusion, these data show that modErk-KTR is highly sensitive to changes in Erk activity, such as in late G2 phase, where Erk target phosphatase activity must be high. Mitotic erasure of P-Erk induces oscillations in Fgf signaling in the presumptive mesendoderm (Figure 7I), and the period and amplitude of these oscillations correlate with distance from the margin, where the Fgf ligand levels are highest. These oscillations are a source of heterogeneity across the signaling gradient.

## Discussion

### An improved Erk-specific biosensor

Here, we have generated a modified Erk-KTR where we have abolished the Cdk1 responsiveness of the original Erk-KTR while maintaining a comparable Erk-specific response in slowly dividing NIH-3T3 cells. We have demonstrated its applicability as an Erk-specific biosensor in highly proliferative and non-proliferative tissues. Our work highlights the importance of validating reporter systems when applying them to new contexts, as a lack of Erk specificity will likely pose a general problem when using Erk-KTR in proliferative tissues. A recent study highlighted the same problem with an Erk FRET reporter and Erk-KTR, where Cdk1-dependent reporter activity increased in late G2 phase in human colorectal cancer cells.^22^ Thus, modErk-KTR will be a valuable tool, providing an improved biosensor for use *in vitro* and *in vivo*.

### P-Erk versus KTR dynamics

Here, we have monitored the formation of the Fgf signaling gradient in the living zebrafish blastula. Although we observed comparative timings with the growth of the P-Erk gradient,^18^ we note that modErk-KTR, in this context, reports on the cumulative dose of Erk activity rather than absolute levels. This is evident in the most marginal cells that experience dampened P-Erk levels yet display the highest KTR C/N ratios. This is explained by their experiencing Erk signaling the longest and the slow rate of modErk-KTR dephosphorylation. It will be important to determine whether this phenomenon is shared by endogenous Erk targets and therefore provides a mechanism of “memory-retention” of past Erk activity.

The rate of modErk-KTR dephosphorylation is much slower (40–70 min) than that of P-Erk (10–20 min) in the early zebrafish embryo, in contrast to more differentiated cells (15–30 min).^44^ This suggests that here there is little/no robust negative feedback downregulating Erk target phosphorylation. We have previously shown that the P-Erk phosphatase, Dusp4, is highly expressed in the first two cell tiers from the margin, and Dusp6 is also broadly expressed throughout the margin. As a result, P-Erk is rapidly lost upon the inhibition of upstream activa-tors.^18,45^ modErk-KTR dephosphorylation must be driven by different phosphatases that are present/active at lower levels during early development but are more highly expressed/active in more differentiated cells. Calcineurin dephosphorylates ELK1 (Sugimoto et al.^46^) and was recently identified in a screen for ERK signaling modulators to regulate ERK-KTR activity.^41^ However, we show that this is not the case in the zebrafish blastula. That study also identified protein phosphatase 2A (PP2A) as a regulator of ERK-KTR localization, although PP2A targets multiple nodes in the mitogen-activated protein kinase (MAPK) pathway (e.g., Raf, Mek, and Erk).^47^ Indeed, the promiscuity of phosphatase catalytic subunits makes it difficult to differentiate direct versus indirect action on targets,^48^ although a broad-acting phosphatase/s would be an attractive candidate for the rapid shutdown of all MAPK pathway activity.

These data highlight the importance of considering how closely linked the phosphorylation/dephosphorylation rates are of the target kinase and its KTR. In the zebrafish blastula, it is unlikely that any interphase Erk dynamics could be observed due to the slow KTR dephosphorylation rate, whereas in other systems, such as mESCs, frequent pulses (8 pulses/h) of ERK activity are registered using the ERK-KTR.^49^ A recent study found that an incoherent feedforward motif could act as a detector of pulsatile signaling, and this was used to generate a synthetic ERK reporter called the READer circuit.^50^ ERK signaling is required to induce the circuit, but ERK must be subsequently turned off for the expression of a fluorescent reporter. Such a circuit highlights how oscillatory signals can encode information and provides the means to further test whether there are additional interphase Erk signaling dynamics in the presumptive mesendoderm.

### Mitotic erasure of Fgf/Erk signaling

By monitoring Erk activity at high temporal resolution (1 min intervals), we find that mitotic erasure of Erk activity and its downstream targets induces oscillations in Fgf/Erk signaling over time (Figure 7I). The consistency of Erk inactivation 2–3 min prior to mitosis suggests a link to the G2-M checkpoint, as similarly reported in zebrafish endothelial cells^11,51^ and skin epithelium,^9^ although the latter was in the context of the Cdk1-responsive Erk-KTR. Erk responses are known to be cell cycle sensitive. For example, G1/S-phases are associated with delayed Erk activation and G2 with rapid sustained signaling in yeast,^52^ whereas mESCs display spontaneous pulses of Erk activity early in the cell cycle.^49^ Although the mechanism of mitotic erasure is currently unknown, the Cdc25 dual-specificity phosphatases are attractive putative regulators as they are active in late G2 phase to induce Cdk1 activity.^53^ Although it is currently unknown whether they dephosphorylate P-Erk targets, Cdc25A can function as an ERK phosphatase in human hepatoma cells.^54,55^

Erk signaling also plays a role in the regulation of progression through G2/M- and G1/S-phases. Inhibition of Erk activity is sufficient to arrest cells in G1-phase and slow the rate of entry into M-phase.^56,57^ Conversely, hyperactivation of Erk can either enhance cell cycle entry or induce cell cycle arrest, depending on signaling levels. Mitotic erasure may therefore play a regulatory role in Erk-dependent cell cycle progression.

Post-mitosis, marginal cells must reactivate Erk signaling, and we observe variability in the reactivation rate that correlates with distance from the margin, suggesting that the reactivation rate is sensitive to ligand availability. Indeed, it is well established that Fgf ligands elicit a concentration-dependent response, both in the amplitude and rate of Erk phosphorylation.^58,59^ Although the heterogeneity can partially be attributed to the animal-marginal movement of cells, there is also clearly cell-autonomous heterogeneity in signal response. During the specification of cranial-cardiac progenitors in the invertebrate chordate, *Ciona intestinalis*, the asymmetric inheritance of internalized FGFRs enables differential sister cell responses to uniformly distributed ligand.^60,61^ This may be a common mechanism for coupling tissue growth and patterning downstream of RTK signaling^35,51,62^ and, whether actively or stochastically driven, could contribute to heterogeneity in Erk signaling in the zebrafish blastula.

Similar to the work described here, we have previously shown there is heterogeneity in P-Erk levels.^19^ We propose that mitotic erasure in combination with cell-autonomous differences in reactivation rates, signal amplitude, and cell cycle asynchrony are all potential sources of noise in Fgf signal interpretation over time (Figure 7I). Two recent studies using ERK-KTR have described a role for FGF/ERK signaling dynamics in early mouse patterning.^7,8^ Simon and coworkers showed that elevated ERK activity promotes primitive endoderm (PrE) specification, whereas epiblast identity was associated with sporadic pulses. Pokrass and coworkers similarly found that following mitosis, FGF/ERK signaling levels diverge, which dictates PrE versus epiblast differentiation through the ERK-dependent destabilization of Nanog, a key epiblast-promoting factor. The authors did not observe mitotic erasure in these studies, likely due to lower temporal resolution. Nevertheless, these results suggest that mitotic erasure of ERK signaling is a conserved process that regulates cell fate decision-making. In the future, it will be interesting to investigate if/how mitotic erasure influences the interpretation of Fgf signaling in the zebrafish blastula and how this might impact embryonic patterning.

### Limitations

When using biosensors that are based on substrates of a kinase of interest (KOI), it is important to establish that the biosensor and KOI activation/deactivation rates are correlated when interpreting reporter output. In zebrafish blastulae, the rate of P-Erk dephosphorylation is much faster than that of modErk-KTR during interphase. Importantly, this is not the case in other contexts, including mitosis. These observations indicate that biosensors in interphase embryonic cells may be less sensitive to rapid Erk dynamics, if they are occurring, due to the stability of Erk-induced target phosphorylation. It is also important, when comparing different cell types, to consider the possibility of differential nuclear import/export rates and cell morphology that will impact the KTR baseline and kinetics. A thorough characterization of the KTR response and context-specific baseline levels is therefore important when translating these reporters to new models. The KTR system alone is not ideal for use in densely packed tissues or those with non-uniformly shaped cells. To overcome this, the use of a co-expressed nuclear marker (e.g., H2Av-mCherry) has been demonstrated to enable the readout of Erk activity based on nuclear fluorescence (Figure 5; Yuen et al.^34^ and de la Cova et al.^35^). It will therefore be useful to generate new zebrafish transgenic lines that utilize this polycistronic system.

## Star★Methods

### Key Resources Table

**Table:** 

Reagent or Resource	Source	Identifier
Antibodies		
Anti-phospho-Smad2 (Zebrafish IF, Dilution: 1:500)	Cell Signaling Technology	Cat # 8828; RRID: AB_2631089
Anti-diphospho-ERK (Zebrafish IF, Dilution: 1:500)	Sigma	Cat # M8159; RRID: AB_477245
Anti-diphospho-ERK (*Drosophila* IF, 1:200)	Cell Signaling Technology	Cat #9101; RRID: AB_331646
Anti-phospho-histone H3 (Zebrafish IF, Dilution: 1:1000)	Abcam	Cat#ab183626
Anti-phospho-histone H3 (Zebrafish IF, Dilution: 1:500)	Cell Signaling Technology	Cat # 9706, RRID: AB_331748
HRP-conjugated anti-mouse secondary antibodies (IF, Dilution: 1:500)	Dako	Cat # P0447 RRID: AB_2617137
HRP-conjugated anti-rabbit secondary antibodies (IF, Dilution: 1:500)	Dako	Cat # P0448 RRID: AB_2617138
Chemicals, peptides, and recombinant proteins		
Tyramide hydrochloride	Sigma	Cat # T2879
NHS-Fluorescein ester	ThermoFisher Scientific	Cat #46410
Cy3 mono NHS ester	Sigma	Cat# PA13101
Cy5 mono NHS ester	Sigma	Cat# PA15101
PD-0325901	Merck	Cat # 444968
RO-3306	Sigma	Cat #217721
Cyclosporin A	Selleck	Cat # S2286
DAPI	Sigma	Cat# 10236276001
FuGene	Promega	Cat # E269A
Fetal Bovine Serum	Thermofisher Scientific	Cat# 10270-106
DMEM/F-12	ThermoFisher Scientific	Cat# 10565018
Halocarbon oil 27	Sigma	Cat # H8773
Halocarbon oil 700	Sigma	Cat # H8898
Critical Commercial Assays		
Multiplex Fluorescent Assay v2	ACDBio	acdbio.com
Experimental models: Cell Lines		
NIH-3T3 cells, mouse	Francis Crick Institute Cell Services	N/A
Experimental models: Organisms/Strains		
Zebrafish *Danio rerio* : WT	Francis Crick Aquatics	N/A
Zebrafish *Danio rerio* : *tg(ubiPErk-KTR-Clover)*	Mayr et al.^9^	N/A
Zebrafish *Danio rerio* : *tg(ubiP:modErk-KTR-Clover)*	This paper	N/A
*Drosophila melanogaster*: P{y[+t7.7] w[+mC]=UAS-ERK-KTR-T2A-H2Av-mCh} attP64/TM3, Sb[1]	Yuen et al.^34^	BDSC:93895
*Drosophila melanogaster*: y[1] w[*];P{y[+t7.7] w[+mC]=UAS-modERK-KTR-T2A-H2Av-mCh}attP40/CyO	This paper	BDSC:95286
*Drosophila melanogaster*: y[1] w[*]; P{y[+t7.7] w[+m*]=nanosP-ERK-KTR-T2A-H2Av-mCh}attP2/TM3, Sb[1]	This paper	N/A
*Drosophila melanogaster*: y[1] w[*]; P{y[+t7.7] w[+m*]=nanosP-modERK-KTR-T2A-H2Av-mCh}attP2/TM3, Sb[1]	This paper	BDSC:95288
Recombinant DNA		
pCS2-*Tol2 recombinase*	Kawakami et al.^63^	N/A
pCS2-*mScarletI-H2B*	This paper	N/A
pCS2-*fgf8a*	van Boxtel et al.^16^	N/A
pCS2- *XdnFGFR*	Amaya et al.^64^	N/A
*pDEST*- *ubiP* : *ERK-KTR-Clover-pA-Tol2*	Mayr et al.^9^	N/A
*pDEST*- *ubiP* : *modErk-KTR-Clover-pA-Tol2*	This paper	N/A
*pUASt* - *modERK-KTR-T2A-H2Av- mCherry-attB*	This paper	N/A
*pNosP* - *ERK-KTR-T2A-H2Av-mCherry-attB*	This paper	N/A
*pNosP* - *modERK-KTR-T2A-H2Av- mCherry-attB*	This paper	N/A
*pCS2-modErk-KTR-Clover*	This paper	N/A
*pCS2-modErk-KTR^AAA^-Clover*	This paper	N/A
*pCS2-modErk-KTR^EEE^-Clover*	This paper	N/A
Software and algorithms		
FIJI (ImageJ)	Schneider et al.^65^	https://imagej.net/Fiji/Downloads
Prism	GraphPad	https://www.graphpad.com/scientific-software/prism/
Other		
Dr*gsc*-C3 (RNAscope)	ACDbio	Cat # 427301-C3
Dr*fgf3*-C4 (RNAscope)	ACDbio	Cat # 850161-C4
Dr*fgf8a*-C2 (RNAscope)	ACDbio	Cat # 559351-C2
35 mm Petri dish, 14 mm microwell No. 1.5 coverglass	MatTek Life Sciences	Cat# P35G-1.5-14-C
lumox® dish with foil base, 0: 50 mm	Sarstedt AG & Co	Cat # 94.6077.305
Coverslip No. 1 18x18mm	Scientific Laboratory Supplies	Cat# MIC3110
Coverslip No. 0 18x18mm	Scientific Laboratory Supplies	Cat# MIC3100
Coverslip No. 1.5 24x40mm	Scientific Laboratory Supplies	Cat # MIC3252

### Resource Availability

#### Lead Contact

Further information and requests for resources and reagents should be directed to and will be fulfilled by the lead contact, Caroline Hill (caroline.hill@crick.ac.uk).

#### Materials Availability

Plasmids and zebrafish lines generated in this study are maintained in the lab by the lead contact, Caroline Hill (caroline.hill@crick.ac.uk) and will be made available upon request. *Drosophila* transgenic lines have been deposited in the Bloomington Drosophila Stock Center.

### Experimental Model And Study Participant Details

#### Zebrafish lines and maintenance

Zebrafish (*Danio rerio*) were housed in 28°C water (pH 7.5 and conductivity 500 μS) with a 15 hr on/9 hr off light cycle. All zebrafish husbandry was performed under standard conditions according to institutional (Francis Crick Institute) and national (UK) ethical and animal welfare regulations. All regulated procedures were carried out in accordance with UK Home Office regulations under project license PP6038402, which underwent full ethical review and approval by the Francis Crick Institute’s Animal Ethics Committee.

#### *Drosophila* lines and maintenance

All experiments were performed in *Drosophila melanogaster* (see key resources table for details of strains used). Flies were grown and maintained at 18°C and during embryo collection they were maintained at 25°C on standard *Drosophila* growth media. Embryos were collected using apple juice agar plates with additional food as above and aged to 2–4 hpf before imaging.

#### Cell culture

NIH-3T3 cells were obtained from Richard Treisman (Francis Crick Institute) and cultured in Dulbecco’s modified Eagle’s medium (DMEM) supplemented with 10% FBS and 1% Penicillin/Streptomycin (Pen/Strep). Cells have been banked by the Francis Crick Institute Cell Services, certified negative for mycoplasma and were species confirmed.

### Method Details

#### Molecular biology and transgenesis

To generate modErk-KTR, the Erk-KTR-Clover sequence from *pDEST*-*ubiP*:*ERK-KTR-Clover-pA-Tol2* (Mayr et al.^9^) was codon-optimized for zebrafish with the following modifications: aga>gct (Arg>Ala) at amino acid 318 (see Figure 3A) and the addition of a C-terminal ‘tttcaattccca’ (FQFP) motif. The modified sequence was subcloned into the BamHI sites of *pDEST*-*ubiP*:*ERK-KTR-Clover-pA- Tol2* to generate *pDEST*-*ubiP*:*modErk-KTR-Clover-pA-Tol2*. Transgenic zebrafish were generated by injecting the plasmid into zebrafish embryos, which was randomly inserted into the genome using Tol2 recombinase-mediated transgenesis.^63^ To generate *pCS2-mScarlet-I-H2B*, *mScarlet-I* was amplified from *pmScarlet-i_C1* (Addgene, # 85044) and *H2B* was amplified from *pCS2-mKeima-H2B* (a gift from Nancy Papalopulu) with an N-terminal GS-linker and inserted into *pCS2* at EcoRI and StuI sites.

To generate modErk-KTR^AAA^ and modErk-KTR^EEE^ expression constructs, modErk-KTR-Clover was first cloned into pCS2 and phospho-acceptor (S/TP) sites serine 45, threonines 57 and 64 were mutated to alanines or glutamates, respectively. Numbering is relative to the beginning of the ELK1 fragment.

The modERK-KTR sequence with T2A-H2Av-mCherry as previously used^34^ was codon-optimized for *Drosophila* and synthesized by Thermo Fisher Scientific GeneArt, then subcloned using EcoRI and SalI into the EcoRI and XhoI sites of *pUASt-attB* (Yuen et al.^34^). The insert was excised from pUASt-attB-modERK-KTR-T2A-H2Av-mCherry and subcloned into the NotI and NheI sites of *pCasper-nosP-HA-brat-attB*, replacing *HA-brat* (a gift from Hilary Ashe). This generated *pUASt*-*modERK-KTR-T2A-H2Av-mCherry-attB*, *pNosP*-*ERK-KTR-T2A-H2Av-mCherry-attB* and *pNosP*-*modERK-KTR-T2A-H2Av-mCherry-attB*. Transgenic flies were generated by injection of the plasmids into fly embryos carrying an attP2 landing site and integrated using ΦC31 integrase. Injections were carried out by BestGene Inc or the Crick Fly Facility. All transgenic lines have been deposited and are available from the Bloomington Drosophila Stock Center, Bloomington, Indiana, USA.

#### mRNA injection of zebrafish embryos

Capped RNA for injection was transcribed using the mMessage mMachine Sp6 or T7 kit (ThermoFisher Scientific) followed by LiCl precipitation. For live imaging experiments, zebrafish embryos were injected with 25 pg *H2B-mScarlet-I* and 400 pg of *modErk-KTR-Clover*, *modErk-KTR^AAA^-Clover* or *modErk-KTR^EEE^-Clover* mRNA at the one-cell stage. For overexpression experiments, embryos were injected with 50 pg *fgf8a* or 500 pg *dnFGFR* mRNA.^16,64^

#### Cell culture

NIH-3T3 cells were grown in a glass bottom 35 mm MaTek dish and transfected with the *pDEST*-*ubiP*:*ERK-KTR-Clover-pA-Tol2* or *pDEST*-*ubiP*:*modERK-KTR-Clover-pA-Tol2* using FuGene (Promega) according to the manufacturer’s instructions. Prior to imaging, cells were incubated in DMEM with 0.5% FBS overnight to ensure baseline ERK activity. ERK activity was induced by addition of 10% FBS.

#### Live imaging

Zebrafish embryos were collected and maintained at 28°C until 3.5 hpf. Embryos were mounted in their chorion in 1% low melting agar (Sigma) on a glass bottom 35-mm MaTek dish and bathed in embryo media (E2 buffer) with or without chemical inhibitors (see below). Embryos were oriented manually to ensure a lateral view and to exclude the dorsal region, which experiences early Fgf/Erk activity at 4 hpf. Embryos were imaged on a Leica SP8 inverted confocal microscope using an HC PL APO CS2 20x/ 0.75 IMM objective at 28 °C with the following confocal settings, pinhole 1 airy unit, scan speed 400 Hz unidirectional, format 512×512 pixels at 8 bit. Images were collected using hybrid detectors and an argon and 561 nm lasers with 2x line averaging and *z*-slices taken at 2 μm intervals every 1 min (Figure 7) or 5 min (Figure 6). Imaging of the dorsal hindbrain at 24 hpf was carried out as described previously.^66^

Live imaging of *Drosophila* embryos was carried out as described.^67^ Embryos were dechorionated in bleach and positioned laterally on top of a coverslip (No. 1, 18 × 18 mm) thinly coated with heptane glue. A drop of halocarbon oil mix (4:1, halocarbon oil 700: halocarbon oil 27)) was placed in the middle of a Lumox imaging dish and two coverslips (Nr. 0, 18 × 18 mm) were placed on either side of the oil drop. The coverslip with the embryos attached was then inverted into the oil, sandwiching the embryos between the imaging dish membrane and the coverslip. Embryos were imaged on a Leica SP8 inverted confocal microscope using an HC PL APO CS2 20x/ 0.75 dry objective at 25°C with the following confocal settings, pinhole 1 airy unit, scan speed 400 Hz unidirectional, format 512 x 512 pixels at 8-bit. Images were collected using hybrid detectors and an argon and 561 nm lasers with 1x line averaging and *z*-slices taken at 2 μm intervals every 3 min.

Live imaging of NIH-3T3 cells was performed as described above on a Leica SP8 inverted confocal microscope using an HC PL APO CS2 20x/ 0.75 IMM objective at 37°C and 10% CO_2_. Images were collected with 2x line averaging and *z*-slices taken at 1 μm intervals every 1.5 min.

#### Zebrafish wounding

Embryos at 48 hpf were immobilized with tricaine (0.08 mg/ml) in E2 buffer and mounted laterally in 1% low melting agar on a glass bottom 35-mm MaTek dish. Wounding was achieved by manually puncturing the muscle with a glass needle.

#### Fluorescence in situ hybridization (FISH) and immunofluorescence (IF)

Combined FISH and IF was performed with the RNAscope® 2.0 Assay using the Multiplex Fluorescent Assay v2 (ACDBio) as previously described^68^ with minor modifications. Briefly, after fixation in 4% paraformaldehyde (PFA), followed by incubation overnight in methanol, embryos were rehydrated and incubated with Dr-*gsc* (427301-C3, ACDBio), Dr-*fgf8a* (559351-C2, ACDBio) and/or Dr-*fgf3* (850161-C4, ACDBio) probes at 40°C overnight. Embryos were then washed in 0.2x saline sodium citrate/0.01% Tween 20 (SSCT) and re-fixed in 4% PFA for 10 min followed by washes with SSCT. First, they were incubated with two drops of the Amp1 and Amp2 solution at 40°C for 30 min and then incubated with two drops of Amp3 at 40°C for 15 min. After an additional washing step, embryos were incubated with two drops of the Multiplex FL V2 HRP-C2, -C3 or -C4 at 40°C for 15 min. After a last series of washes in SSCT, embryos were washed in PBS/ 0.1% Tween-20 (PTW) and processed for the staining. Like conventional FISH, embryos were incubated with tyramide (Sigma) coupled with fluorescein-NHS ester (Thermo Scientific, #46410), Cy3 mono NHS ester (Sigma, #PA13101) or Cy5 mono NHS ester (Sigma, #PA15101) in PTW in the dark. To allow HRP detection, 0.001% H_2_O_2_ was added to the reaction and embryos were incubated for 30 min, also in the dark. The embryos were then extensively washed in PBS/1% Triton X-100 (PBTr) and incubated in acetone at -20°C. After that, embryos were incubated for 2 hr in PBTr with 10% FBS before incubation with antibodies against P-Erk overnight at 4°C. Antibody binding was detected with HRP-conjugated anti-mouse secondary antibodies and signal was developed as above for RNA detection.

IF for P-Smad2, P-Erk and P-H3 was performed as described^18^ with minor modifications. Embryos were rehydrated into PBTr before incubating in acetone at -20°C. Embryos were blocked in 1% PBTr and 10% FBS, before incubating with antibodies against pSmad2 (Cell Signaling Technology, # 8828, 1:500), P-Erk (Sigma, M8159, 1:500) and P-H3 (CST, # 9706, 1:500; or Abcam, ab183626, 1:1000) at 4°C overnight. Antibody binding was detected as above.

In all cases, zebrafish embryos were extensively washed and DAPI was used at 1:1000 in PTW for 15 min at room temperature. Embryos were then mounted in 1% low melting agarose on a glass bottom 35-mm MaTek dish and manually oriented.

For the IF of *Drosophila* eye imaginal discs, wandering 3^rd^ instar larvae were dissected in Schneider’s insect medium (ThermoFisher, # 21720-024) and incubated in 10 μM EdU (5-ethynyl-2′-deoxyuridine) in Schneider’s medium while shaking for 30 min. After incubation, samples were fixed for 15 min in 4% paraformaldehyde in 10 mM Tris-HCl (pH 6.8), 180 mM KCl, 50 mM NaF, 10 mM NaVO_4_, and 10 mM β-glycerophosphate, then washed twice in 0.5% PBTr for 30 min. Samples were blocked in 0.2% PBTr and 1% FBS for 1 hour, then incubated overnight at 4°C in rabbit anti-phospho-ERK antibody (Cell Signaling Technology, # 9101, 1:200). The samples were then washed twice for 30 min in 0.5% PBTr and 1% FBS and subsequently incubated in secondary antibody for 2 hrat room temperature, before being washed in 0.2% PBST for 30 min, then incubated for 30 minutes in 2.5 μM AZ dye 405 picolyl azide (Click Chemistry Tools), 0.1 mM THPTA, 2 mM sodium ascorbate, and 1 mM CuSO_4_. Finally, the samples were washed twice in 0.2% PBTr for 15 minutes and mounted on microscope slides with Vectashield medium (H-1000, Vector labs).

For imaging *Drosophila* ovaries, adult females were raised with males for 3–7 days post-eclosion prior to dissection in order to promote normal reproductive health. Ovaries were dissected in Schneiders insect medium and fixed in 4% PFA for 15 min. They were extensively washed in 0.1% PBTr and DAPI was used at 1:1000 for 15 min at room temperature. Ovaries were mounted on a microscope slide in Prolong Gold Antifade.

All FISH and IF samples were imaged on a Leica SP8 inverted confocal microscope using either a HC PL APO CS2 20x/0.75 DRY objective or 10x DRY objective. Imaginal discs were imaged on a Zeiss LSM880 with a 40X objective.

#### Pharmacological inhibitors

For drug treatments, the inhibitors PD-0325901, RO-3306 and CsA were dissolved in DMSO and directly diluted in embryo or cell culture medium at 10 μM (PD-0325901 and CsA) and 20 μM (RO-3306) respectively. Embryos were maintained at 28°C and the time of treatment and durations are specified in the Figure legends.

### Quantification And Statistical Analysis

#### Image analysis

To quantify single cell Erk activity (Figures 2, 3, and 4), a 5–6 pixel width region of interest was drawn in the centre and periphery in Fiji^65^ to measure the nuclear and cytoplasmic mean intensities, as illustrated in Figure 2A. These were used to calculate the log_2_(cytoplasmic/nuclear) to give a linear readout of Erk activity. To track cells pre- and post-mitosis, H2B-mScarlet-I nuclear signal was used to track single cells manually and XY coordinates were also measured relative to the margin (Y = 0 μm).

To quantify Erk activity across the entire Fgf signaling gradient, a lateral view of the embryo was oriented relative to the margin (Y = 0 μm) and region of interest is drawn to exclude the EVL. H2B-mScarlet-I was used to generate a nuclear mask with unique identifiers using CLIJ.69 This was performed on 4–5 single *z*-slices at 15–20 μm intervals to capture up to 300 μm from the embryonic margin whilst ensuring no overlap between slices. The nuclear mask was dilated by 2 pixels and the original nuclear mask subtracted to generate a cytoplasmic mask with the same ID. The nuclear and cytoplasmic masks were then used to measure mean intensity and XY coordinates. Cells were then grouped into either 20 or 25 μm bins, depending on the stage of development, to determine the number of cell tiers away from the margin. In the brain, nuclei were too closely clustered and therefore Erk activity was manually measured as above.

To quantify P-Erk levels, DAPI was used to generate a nuclear mask and measure P-Erk and DAPI intensity as well as XY coordinates in Fiji. P-Erk levels are presented relative to DAPI intensity and presented relative to the margin as above.

Cell width was measured by manually drawing a line across the centre of cells in the animal-margin axis using the line drawing tool in Fiji.

Analysis of *Drosophila* imaginal discs was carried out in Icy (v2.4.2.0). For each cell, the focal plane containing the largest nuclear diameter was identified. Using the freehand ROI tool, an approximate outline of the nucleus was drawn using the H2Av-mCherry signal. In areas where nuclei were closely packed, adjacent focal planes were used to determine the most suitable ROIs while avoiding overlapping pixels with adjacent cells. The mean intensities of each channel were then measured for each cell and used to obtain the mCherry:Clover ratio as a readout of KTR activity.

#### Statistical Analysis

Statistical comparisons were performed using two-tailed Student’s t-tests, one-way ANOVA with multiple comparisons or paired t-test as indicated in the figure legends using GraphPad Prism and Microsoft Excel. Statistical significance was assumed by p < 0.05. Individual p values are indicated, and data are represented by the mean and standard deviation unless otherwise specified. A linear regression in JMP was used for statistical analysis and fitting a line to the imaginal disc data.

## Supplementary Material

Figure S1

Video S1

Video S2

Video S3

Video S4

Video S5

Video S6

Video S7

## Data Availability

This paper does not report new datasets or any original code. Any additional information required to reanalyse the data reported in this paper is available from the lead contact upon request.

## Highlights

- Erk-KTR displays Cdk1-dependent activity that impairs Erk-specific readout
- modErk-KTR represents an improved Erk-specific biosensor in zebrafish and *Drosophila*
- Mitosis introduces periods of Erk inactivity in zebrafish and *Drosophila* embryos
- Mitotic erasure introduces heterogeneity to Fgf signal interpretation

## In brief

Wilcockson et al. describe the development of an improved Erk-specific kinase translocation reporter (modErk-KTR). Live imaging of Erk dynamics in zebrafish and *Drosophila* embryos shows that Erk signaling is shut down prior to mitosis (mitotic erasure), which contributes to heterogeneity in Fgf signal interpretation in zebrafish embryos.
