# Supplementary material for: An improved Erk biosensor detects oscillatory Erk dynamics driven by mitotic erasure during early development
Source: Dev Cell. Author manuscript; Available in PMC 2023 Dec 4. (PMC7615346; doi:10.1016/j.devcel.2023.08.021)
Supplement: Figure S1 [file EMS189028-supplement-Figure_S1.pdf]

Developmental Cell, Volume *58*

Supplemental information

An improved Erk biosensor detects oscillatory Erk dynamics driven by mitotic erasure during early development

Scott G. Wilcockson, Luca Guglielmi, Pablo Araguas Rodriguez, Marc Amoyel, and Caroline S. Hill

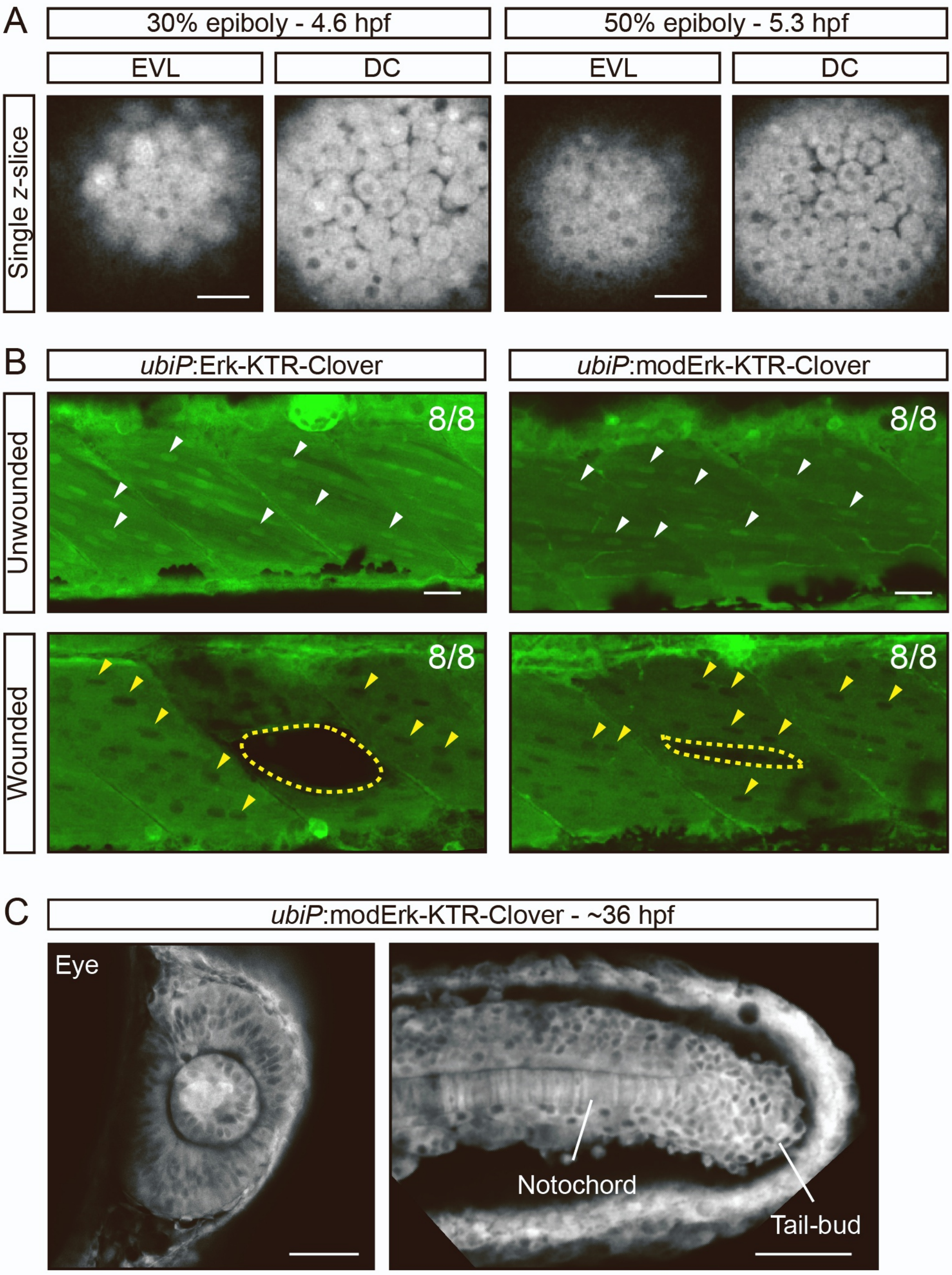
A
30% epiboly - 4.6 hpf
50% epiboly - 5.3 hpf
EVL
DC
EVL
DC
Single z-slice
B
ubiP:Erk-KTR-Clover
ubiP:modErk-KTR-Clover
Unwounded
8/8
8/8
Wounded
8/8
8/8
C
ubiP:modErk-KTR-Clover - ~36 hpf
Eye
Notochord
Tail-bud

Figure S1

Figure S1. Wounding-induced Erk signaling is readout by Erk-KTR and modErk-KTR

(A) Single *z*-slices showing Erk-KTR activity in the enveloping layer (EVL) and deep cells (DCs) from the indicated embryos shown in Figure 4B.

(B) Representative images of 48-hpf *ubiP*:Erk-KTR-Clover and *ubiP*:modErk-KTR-Clover zebrafish embryos with and without muscle wounding. Muscle cells typically display no Erk activity (white arrowheads). However, upon wounding the muscle the surrounding cells rapidly (~15 min) display high Erk activity (yellow arrowheads). Dashed line labels site of wound.

(C) Representative images of *ubiP*:modErk-KTR-Clover zebrafish embryos in other contexts where Fgf/Erk signaling is well characterized, including the developing eye and presomitic mesoderm. Erk activity is not observed in the notochord.

Scalebars, 20 μm (A, B) or 50 μm (C).

Related to Figure 4.

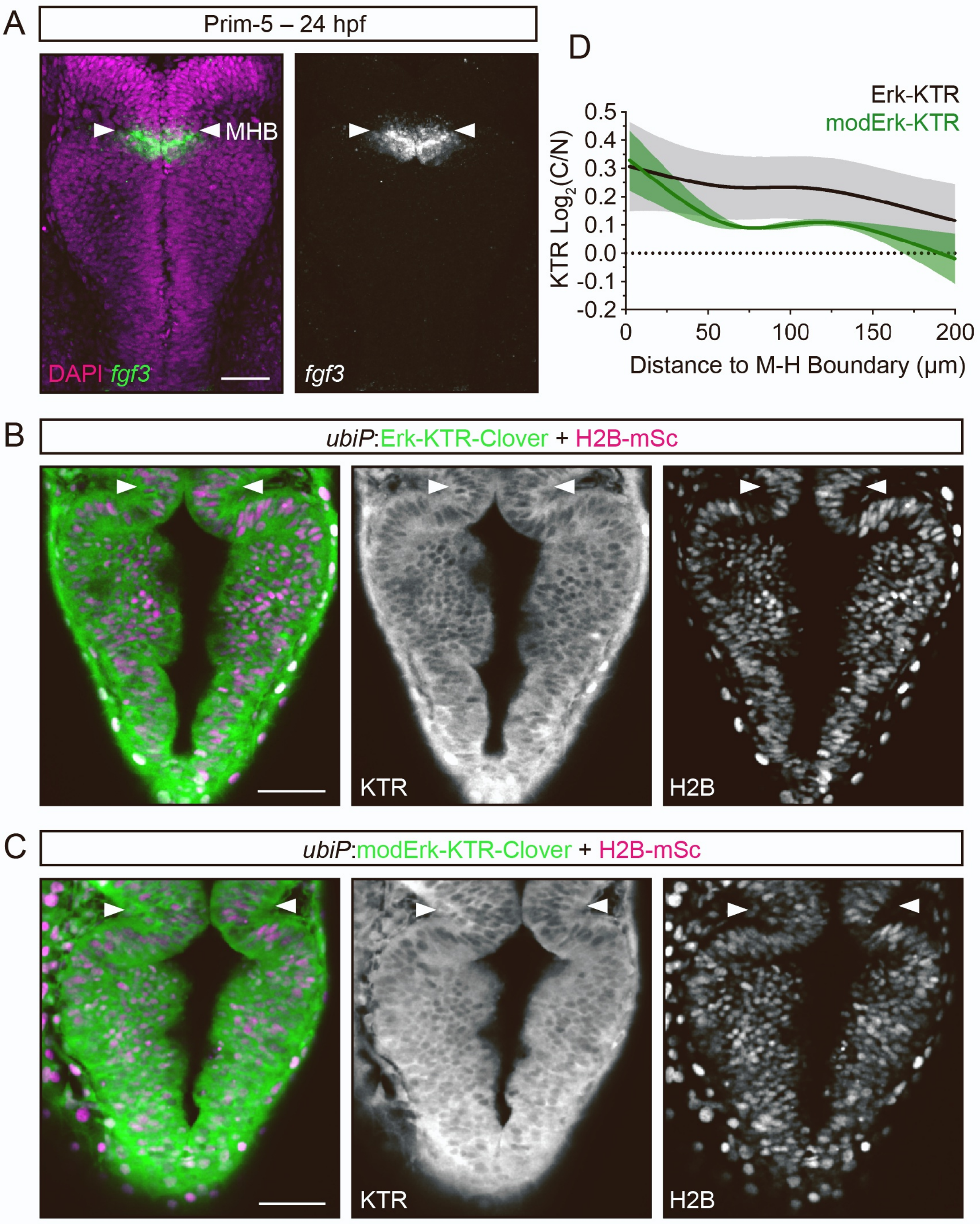
A
Prim-5 – 24 hpf
MHB
DAPI fgf3
fgf3
D
Erk-KTR
modErk-KTR
KTR Log2(C/N)
0.5
0.4
0.3
0.2
0.1
0.0
-0.1
-0.2
0
50
100
150
200
Distance to M-H Boundary (μm)
B
ubiP:Erk-KTR-Clover + H2B-mSc
KTR
H2B
C
ubiP:modErk-KTR-Clover + H2B-mSc
KTR
H2B

Figure S2

Figure S2. Visualization of an Fgf/Erk signaling gradient at the zebrafish midbrain-hindbrain boundary.

(A) RNAscope showing expression of *fgf3* at the midbrain-hindbrain boundary (MHB; white arrows) of pharyngula embryos at 24 hpf (Prim-5 stage).

(B, C) Representative images showing a single plane through the dorsal midbrain of a *ubiP*:Erk-KTR-Clover (C) and a *ubiP*:modErk-KTR-Clover (D) zebrafish embryo, additionally expressing H2B-mScarletI (H2B-mSc). White arrows, MHB.

(D) Quantification of the levels of Erk activity relative to distance from the MHB (n = 3 embryos each; mean ± SD).

Scalebars, 50 μm.

Related to Figure 4 and Figure S1.

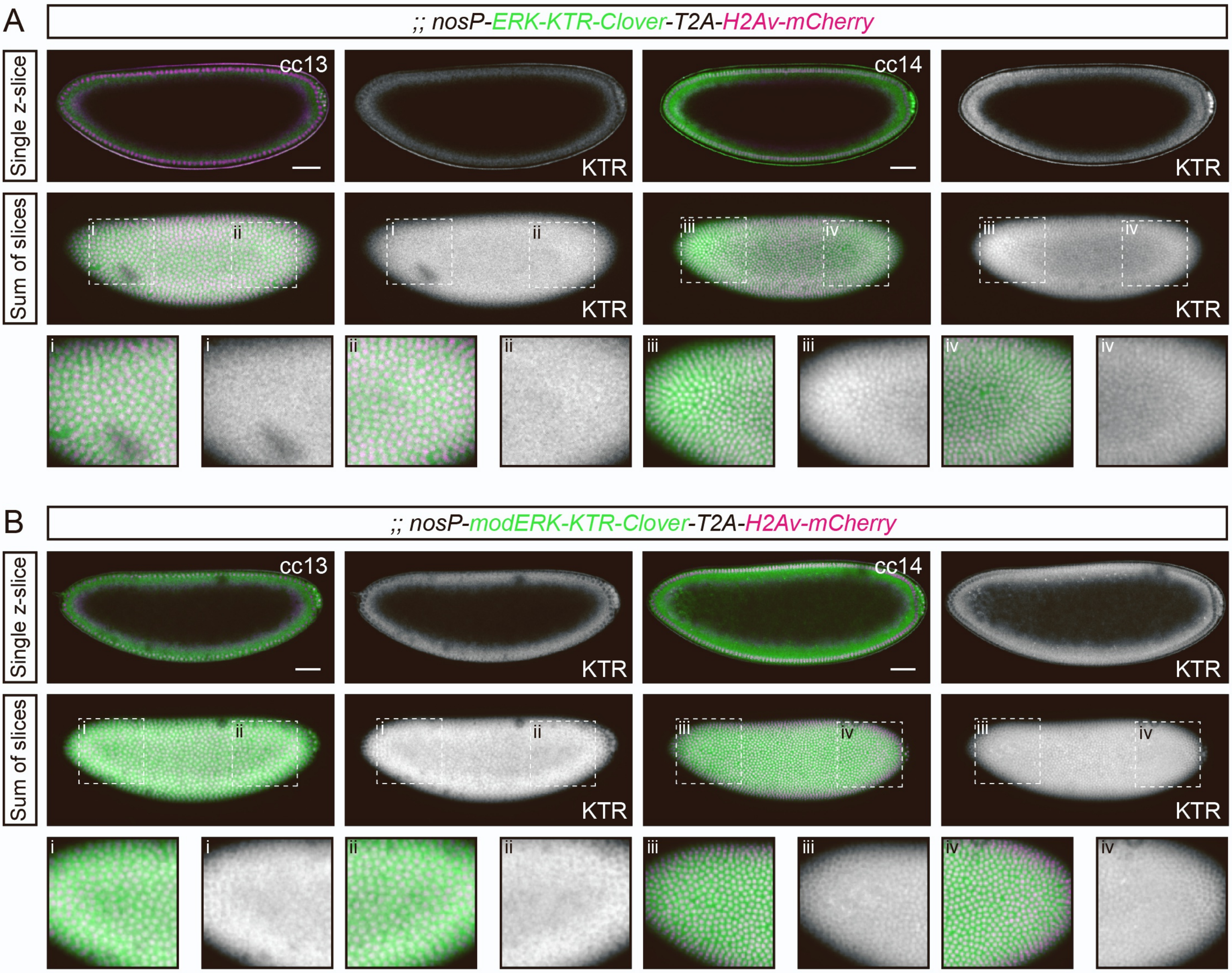
A
;; nosP-ERK-KTR-Clover-T2A-H2Av-mCherry
Single z-slice
Sum of slices
cc13
cc14
KTR
i
ii
iii
iv
B
;; nosP-modERK-KTR-Clover-T2A-H2Av-mCherry
Single z-slice
Sum of slices
cc13
cc14
KTR
i
ii
iii
iv

Figure S3

Figure S3. Comparison of anteroposterior Torso/Erk signaling readout in *Drosophila* embryos.

(A and B) Whole embryo views of the images in Figure 5B and C showing the readout of Erk activity by the original ERK-KTR (A) and modERK-KTR (B) reporters during cell cycles (cc) 13 and 14. Shown are both (top) a single *z*-slice through the centre of the embryo and (middle) a sum of slices projection of the top half of the same embryo. Expanded views of regions within white boxes is also shown (bottom) with (left) and without (right) H2Av-mCh fluorescence to enable visualization of nuclei.

Related to Figure 5.

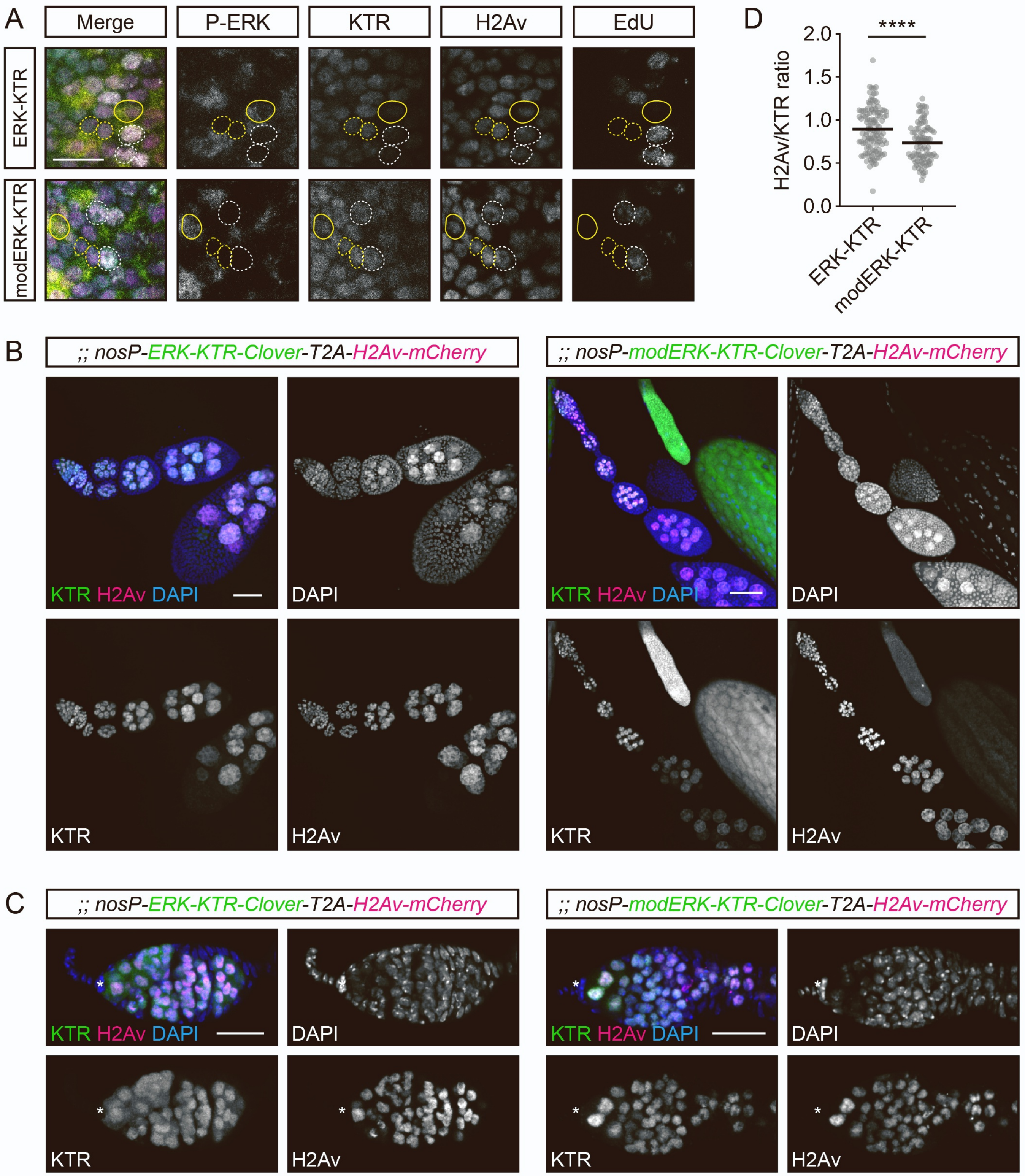
A
Merge
P-ERK
KTR
H2Av
EdU
ERK-KTR
modERK-KTR
D
2.0
1.5
1.0
0.5
0.0
****
H2Av/KTR ratio
ERK-KTR
modERK-KTR
B
;; nosP-ERK-KTR-Clover-T2A-H2Av-mCherry
;; nosP-modERK-KTR-Clover-T2A-H2Av-mCherry
KTR H2Av DAPI
DAPI
KTR
H2Av
C
;; nosP-ERK-KTR-Clover-T2A-H2Av-mCherry
;; nosP-modERK-KTR-Clover-T2A-H2Av-mCherry
KTR H2Av DAPI
DAPI
KTR
H2Av

Figure S4

Figure S4. Comparison of the KTR constructs in the ovarian germline.

(A) Immunofluorescence images of P-ERK levels in both KTR constructs in eye imaginal discs. Dashed lines mark P-ERK negative cells that are either EdU positive (white) or negative (yellow). Yellow lines mark P-ERK positive cells.

(B) Representative images of fixed transgenic *Drosophila* ovarioles stained with DAPI to visualize the somatic tissue. *nosP* drives expression throughout the ovarian germline.

(C) Representative images of fixed germaria as in (B).

(D) Quantification of (C) comparing KTR (quantitated as the H2Av/KTR ratio) read out in germ cells of the anterior germarium. n = 95–118 cells, n ≥ 6 germaria. The black lines show the means.

Scalebars, 10 μm (A), 50 μm (B) or 20 μm (C). Unpaired t test; ****, $p < 0.0001$.

Related to Figure 5.

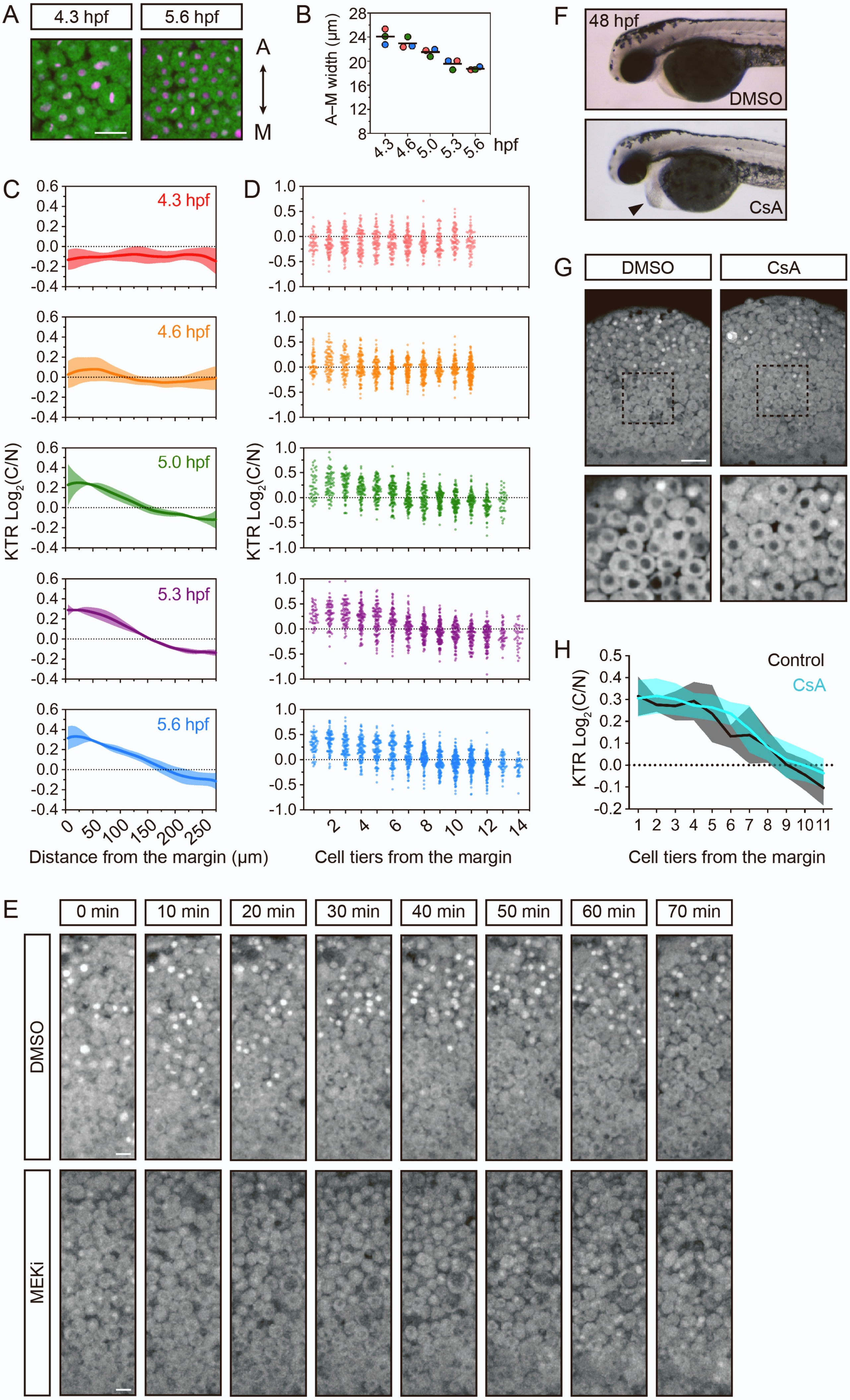
A
4.3 hpf
5.6 hpf
A
M
B
A–M width (μm)
hpf
C
D
4.3 hpf
4.6 hpf
5.0 hpf
5.3 hpf
5.6 hpf
KTR Log2(C/N)
Distance from the margin (μm)
Cell tiers from the margin
E
0 min
10 min
20 min
30 min
40 min
50 min
60 min
70 min
DMSO
MEKi
F
48 hpf
DMSO
CsA
G
DMSO
CsA
H
Control
CsA
KTR Log2(C/N)
Cell tiers from the margin

Figure S5

Figure S5. Interpreting the Fgf/Erk signaling gradient and heterogeneity in signaling levels

(A) Representative images of a single *ubiP*:modErk-KTR-Clover zebrafish embryo injected with 25 pg *H2B-mScarlet-I* mRNA at the one-cell stage and imaged at the indicated times of development. Embryos were imaged laterally and oriented for measuring cell width in the animal–margin (A–M) plane.

(B) Quantification of A–M width from (A) at the indicate times during development. n = 40 cells per embryo, n = 3 embryos per timepoint, black line shows the mean.

(C) Quantification of Erk activity (Log_2(C/N)) in the lateral region of *ubiP*:modERK-KTR-Clover embryos at 20 min intervals from dome (4.3 hpf) to germ ring stage (5.6 hpf), relating to Figure 6B. Erk activity is shown relative to distance from the embryonic margin, mean ± SD.

(D) Quantification of Erk activity as in Figure 6B, showing all data points.

(E) Representative images of lateral views of *ubiP*:modErk-KTR-Clover zebrafish embryos imaged at 5-min intervals following addition of control (DMSO) or 10 μM PD-0325901 (MEKi).

(F) Images of 48 hpf zebrafish larvae treated for 24 hrs with either control (DMSO) or 10 μM CsA. Arrowhead indicates cardiac oedema.

(G) Representative images of lateral views of 5.3 hpf *ubiP*:modErk-KTR-Clover zebrafish embryos following treatment with either control (DMSO) or 10 μM CsA from 4.0 hpf. Expanded single *z*-slice views of the regions within black boxes are shown below.

(H) Quantification of Erk activity (Log_2(C/N)) in (G), means ± SD.

Scalebars, 20 μm.

Related to Figure 6.

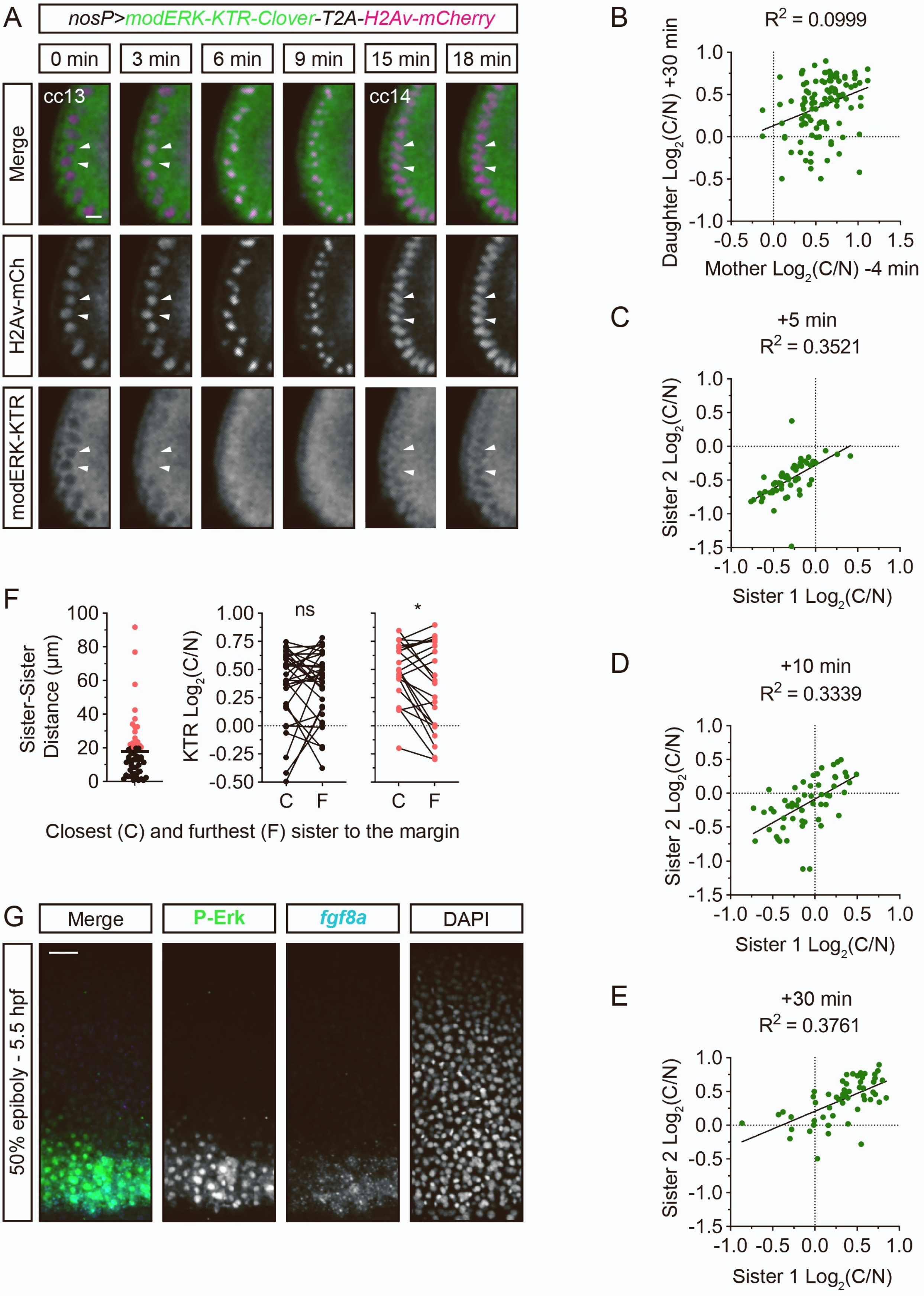
A
nosP>modERK-KTR-Clover-T2A-H2Av-mCherry
0 min
3 min
6 min
9 min
15 min
18 min
cc13
cc14
Merge
H2Av-mCh
modERK-KTR
B
R2 = 0.0999
Daughter Log2(C/N) +30 min
Mother Log2(C/N) -4 min
C
+5 min
R2 = 0.3521
Sister 2 Log2(C/N)
Sister 1 Log2(C/N)
D
+10 min
R2 = 0.3339
E
+30 min
R2 = 0.3761
F
Sister-Sister Distance (µm)
KTR Log2(C/N)
ns
*
C
F
Closest (C) and furthest (F) sister to the margin
G
Merge
P-Erk
fgf8a
DAPI
50% epiboly - 5.5 hpf

Figure S6

Figure S6. Heterogeneity in sister cell Erk activity

(A) Time course showing the anterior pole of a *nosP>modERK-KTR-Clover-T2A-H2Av-mCherry Drosophila* embryo. The embryo was imaged every 3 min and Erk activity monitored around mitosis. White arrowheads label the first two cells to undergo mitosis.

(B) Quantification of the levels of Erk activity in the mother cell (n = 55) at -4 min pre-mitosis and daughter cell (n = 110) at +30 min post-mitosis in blastula/early gastrula stage zebrafish embryos and fitted with a simple linear regression.

(C–E) Quantification of sister cell Erk activity levels at +5 min (C), +10 min (D) and +30 min (E) post-mitosis in blastula/early gastrula stage zebrafish embryos and fitted with a simple linear regression.

(F) Quantification of the final distance between sisters +30 min post-mitosis in the animal-marginal plane. (Left) Sisters that have drifted at least a single cell tier away (≥ 20 µm) (red) were compared to those that remain in proximity (black). (Right) Pair-wise comparison of Erk activity between the sister closest to the margin (C) and the sister furthest from the margin (F) for the different groups of sisters. Paired t test; ns, non-significant (p = 0.5501); *, p = 0.0173.

(G) Combined immunofluorescence and RNAscope showing a P-Erk and *fgf8a* expression at the zebrafish embryonic margin at 5.5 hpf. These are zooms of the images shown in Figure 1C.

Scalebars, 10 µm (A) or 50 µm (G).

Related to Figure 7

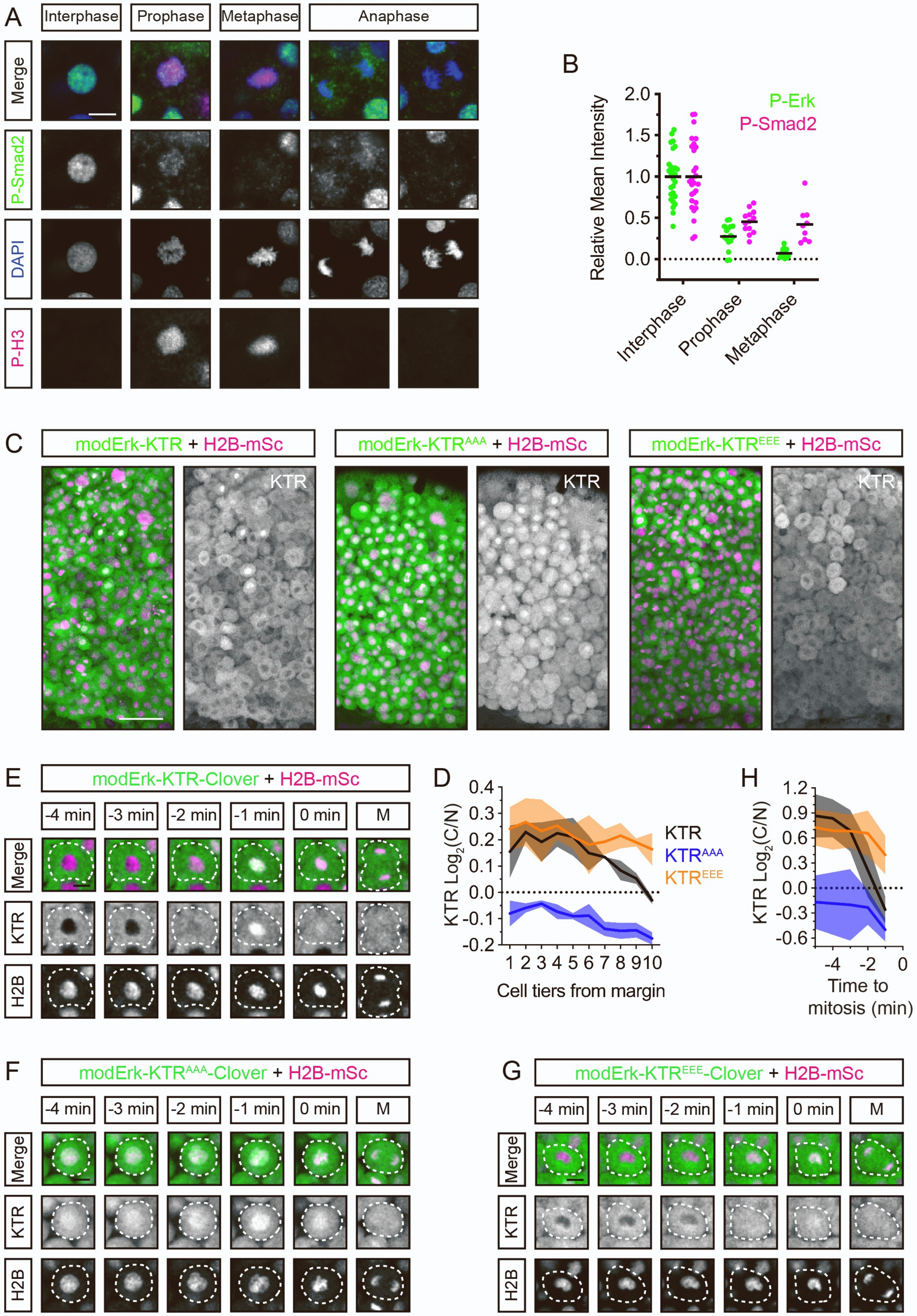

A
Interphase
Prophase
Metaphase
Anaphase
Merge
P-Smad2
DAPI
P-H3
B
Relative Mean Intensity
2.0
1.5
1.0
0.5
0.0
P-Erk
P-Smad2
Interphase
Prophase
Metaphase
C
modErk-KTR + H2B-mSc
modErk-KTRAAA + H2B-mSc
modErk-KTREEE + H2B-mSc
KTR
E
modErk-KTR-Clover + H2B-mSc
-4 min
-3 min
-2 min
-1 min
0 min
M
Merge
KTR
H2B
D
KTR Log2(C/N)
0.4
0.3
0.2
0.1
0.0
-0.1
-0.2
1 2 3 4 5 6 7 8 9 10
Cell tiers from margin
KTR
KTRAAA
KTREEE
H
KTR Log2(C/N)
1.2
0.9
0.6
0.3
0.0
-0.3
-0.6
-4
-2
0
Time to mitosis (min)
F
modErk-KTRAAA-Clover + H2B-mSc
G
modErk-KTREEE-Clover + H2B-mSc

Figure S7

Figure S7, Mitotic erasure of KTR is due to loss of Erk activity

(A) Representative immunofluorescence images of P-Smad2 and P-H3 in zebrafish embryos (4.6 hpf).

(B) Quantification of P-Erk and P-Smad2 levels in (A) and Figure 7D. P-Erk (interphase, n = 27; prophase, n = 16; metaphase, n = 14) and P-Smad2 (interphase, n = 28; prophase, n = 13; metaphase, n = 9). The black lines show the means.

(C) Representative images of lateral views of 5.3 hpf zebrafish embryos injected with 25 pg *H2B-mScarlet-I* mRNA and 400 pg of either *modErk-KTR-Clover*, *modErk-KTR^{AAA}-Clover* or *modErk-KTR^{EEE}-Clover* mRNA at the one cell stage.

(D) Quantification of Erk activity (Log_2(C/N)) of embryos in (C) relative to distance from the margin. n = 3 (KTR) or 4 (KTR^{AAA} and KTR^{EEE}) embryos; mean ± SD.

(E–G) Representative images of a single mesendodermal cell approaching mitosis. Embryos were injected with 25 pg *H2B-mScarlet-I* mRNA and (E) 400 pg of either *modErk-KTR-Clover*, (F) *modErk-KTR^{AAA}-Clover* or (G) *modErk-KTR^{EEE}-Clover* mRNA at the one cell stage and a lateral region of the margin was imaged from ~4.6 hpf at 1 min intervals. White dashed line labels the single cell.

(H) Quantification of Erk activity (Log_2(C/N)) of single cells in (E–G) relative to distance from the margin. n = 10 (KTR), 20 (KTR^{AAA}) or 22 (KTR^{EEE}) cells; mean ± SD.

Scalebars, 10 μm (A, E–G) or 50 μm (C).

Related to Figure 7
